# Structure of the tilapia lake virus nucleoprotein bound to RNA

**DOI:** 10.1093/nar/gkaf112

**Published:** 2025-02-24

**Authors:** Benoît Arragain, Martin Pelosse, Karine Huard, Stephen Cusack

**Affiliations:** European Molecular Biology Laboratory, EMBL Grenoble, 71 Avenue des Martyrs, CS 90181, 38042, Grenoble Cedex 9, France; European Molecular Biology Laboratory, EMBL Grenoble, 71 Avenue des Martyrs, CS 90181, 38042, Grenoble Cedex 9, France; European Molecular Biology Laboratory, EMBL Grenoble, 71 Avenue des Martyrs, CS 90181, 38042, Grenoble Cedex 9, France; European Molecular Biology Laboratory, EMBL Grenoble, 71 Avenue des Martyrs, CS 90181, 38042, Grenoble Cedex 9, France

## Abstract

Tilapia Lake virus (TiLV) belongs to the *Amnoonviridae* family within the *Articulavirales* order of segmented negative-strand RNA viruses and is highly diverged from more familiar orthomyxoviruses, such as influenza. The viral nucleoprotein (NP), a key component of the replication machinery, packages the viral genome into protective ribonucleoprotein particles. Here we describe the electron cryo-microscopy (cryo-EM) structure of TiLV-NP bound to RNA within *in vitro* reconstituted, small ring-like, pseudo-symmetrical oligomers. Although TiLV-NP is considerably smaller than its influenza counterpart and unrelated in sequence, it maintains the same topology and domain organisation. This comprises a head and body domain between which is a positively charged groove, where single-stranded RNA binds. In addition, an oligomerisation loop inserts into a hydrophobic pocket in the neighbouring NP, the flexible hinges of which allow variable orientation of adjacent NPs. Focused cryo-EM maps unambiguously define the 5′ to 3′ direction of the bound RNA, confirmed by double stranded, A-form RNA regions that extrude out from some of the NP–NP interfaces. This is the first fully resolved description of how single-stranded and stem-loop RNA binds to an articulaviral NP assembly. Superposition with orthomyxoviral NPs suggest that the mode of RNA binding is likely similar across the *Articulavirales* order.

## Graphical Abstract

**Graphical Abstract:**
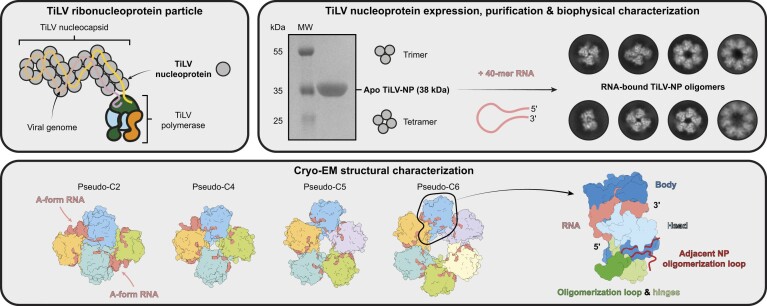

## Introduction

Tilapia Lake virus (TiLV) is a recently discovered pathogen of tilapia fish that can cause mass dieoffs in commercial fish farms [1]. It has now been detected throughout the tropics and in the USA and represents a significant threat to a globally important proteinaceous food source [2]. TiLV belongs to the *Amnoonviridae* family (https://ictv.global/taxonomy/taxondetails?taxnode_id=202306025&taxon_name=Amnoonviridae.) within the *Articulavirales* order of segmented negative-strand RNA viruses (sNSV). It has 10 single-stranded RNA genome segments, each with conserved, quasi-complimentary 3′ and 5′ ends, a characteristic of sNSV [3]. The genome segments encode putative viral proteins whose sequences show no homology to any other protein apart from that of segment 1, which has the conserved motifs characteristic of an orthomyxovirus PB1-like polymerase core subunit [3]. A recent structural analysis showed that the complete, functional TiLV RNA-dependent RNA polymerase is a heterotrimer of proteins encoded by segments 1, 2, and 3 [4]. Despite being 40% smaller in size and totally diverged in sequence, TiLV polymerase has an architecture similar to influenza polymerase and contains minimised versions of most of the corresponding domains. Furthermore, TiLV polymerase binds the conserved 3′ and 5′ viral or complementary RNA (vRNA or cRNA) ends in a similar fashion to the influenza viral promoter, with a 5′ hook and distal duplex region, in either mode A (with the 3′ end in the polymerase active site) or mode B (with the 3′ end in the secondary binding site). Finally, recombinant TiLV polymerase can take up distinct ‘transcriptase’ and ‘replicase’ conformations and actively synthesises RNA *in vitro* [4].

The other key component of the sNSV replication machinery is the viral nucleoprotein (NP). Multiple copies of NP package the vRNA or cRNA into ribonucleoprotein particles (RNPs), which are the functional templates for transcription and replication. Orthomyxovirus RNPs are flexible rods in which NP-bound RNA is arranged in two anti-parallel strands with an overall super-helical twist. The polymerase binds to the 3′ and 5′ (anti-)genomic extremities at one end, and there is an RNA loop at the other end [5]. The RNPs are necessarily flexible and dynamic, since RNA with potentially secondary structure has to be accommodated and NPs have to be successively stripped off the template and then replaced during RNA synthesis, preserving RNP integrety [6, 7]. Encapsidation of nascent genome replicates is a complex process thought to be mediated by the replication complex, an asymmetric dimer, which, for influenza, is composed of two viral polymerases bridged by host factor ANP32 [8–10]. ANP32 is proposed to promote co-replicational encapsidation of the elongating replicate within the replication complex by direct binding to and recruitment of successive NPs, thus growing the progeny RNP [11].

The high resolution structure of apo-NP is known for several orthomyxoviruses including influenza A [12–15], influenza B [16], influenza D [17], Thogoto (THOV) [18] and infectious salmon anaemia (ISAV) [19]. Orthomyxovirus NPs exhibit a common fold and NP–NP interaction mechanism, but the inter-subunit flexibility and RNA sequence heterogeniety has so far only allowed low-resolution structure determination of the native RNPs from influenza [7, 20, 21] or THOV [18]. Indeed, the handedness of the RNP helix remains controversial, with studies describing it as right handed [21, 22] or left handed for influenza [7], and left handed for THOV [18]. Furthermore, the mode and direction of binding of the RNA to NP has been hitherto unknown. However, whilst this manuscript was in review, a high resolution structure of *in vitro* reconstituted right handed, anti-parallel, double helical influenza RNP-like assembly was published [23], which provides the first detailed description of RNA binding to influenza A NP (see ‘Discussion’).

For TiLV, a lack of sequence homology precluded straightforward identification of the segment encoding a putative NP. However, a previous study has provided convincing experimental evidence that segment 4 encodes the TiLV-NP [24]. The protein was shown to bind in multiple copies to RNA in infected cells and RNP-like particles were immuno-purified from infected cells and virions.

Here we express in insect cells and purify TiLV-NP and biophysically characterise its oligomeric states in apo form and in complex with a 40-mer RNA. By electron cryo-microscopy (cryo-EM), we determine its structure bound to RNA, in the context of small pseudo-symmetrical tetramers, pentamers, and hexamers. Despite flexibility within the particles, the resolution is sufficient to unambiguously define the TiLV-NP structure and the mode and directionality of RNA binding. Although smaller than the influenza NP (354 residues compared to 498 for FluA/NP) and with no sequence homology, the topology of the TiLV-NP fold resembles that of orthomyxoviruses. The implications for RNP structure and dynamics are discussed.

## Material and methods

### Cloning, expression and purification of the TiLV-NP

As previously described [4], the 10 TiLV open reading frames, codon-optimised for insect cell expression (Genscript), were subcloned into multiple pFastBac Dual vectors using EcoRI and SpeI. TiLV segment 4, encoding TiLV-NP was amplified and inserted into a psLIB plasmid with an N-terminal deca-poly-histidine tag followed by a Tobacco Etch Virus (TEV) protease cleavage site, using a combination of polymerase chain reaction and Gibson Assembly (NEB).

The EMBacY bacmid containing the TiLV-NP gene was prepared using the Bac-to-Bac method (Invitrogen) and subsequently used for insect cell expression. For large-scale expression, *Trichoplusia ni* High 5 cells (ThermoFisher) at a concentration of 0.8–1 × 10^6^ cells/mL were infected by adding 1% of the virus. Expression was stopped 72–96 h after the day of proliferation arrest, and the cells were harvested by centrifugation (1000 g, 20 min, 4°C). The cells were disrupted by sonication for 3 min (10 s ON, 20 s OFF, 40% amplitude) on ice in lysis buffer (50 mM HEPES pH 8, 500 mM NaCl, 20 mM imidazole, 0.5 mM TCEP, and 5% glycerol) with cOmplete EDTA-free Protease Inhibitor Cocktail (Roche). After lysate centrifugation at 48 000 g for 45 min at 4°C, the soluble fraction was loaded on a HisTrap HP ion affinity chromatography column (Cytiva). Bound proteins were subjected to two sequential wash steps using (i) the lysis buffer supplemented with 1 M NaCl and (ii) the lysis buffer supplemented with 50 mM imidazole. Bound proteins were eluted gradually from 50 to 500 mM imidazole over 15 column volumes.

TiLV-NP fractions were pooled. TEV protease was added for His-tag cleavage (1:50 w/w ratio), and the protein mixture was dialyzed overnight at 4°C in a heparin-loading buffer (50 mM HEPES pH 8, 250 mM NaCl, 0.5 mM TCEP, 5% glycerol). Proteins were loaded on a HiTrap Heparin HP column (Cytiva), washed using the heparin-loading buffer, and eluted gradually from 250 mM NaCl to 1 M NaCl, over 15 column volume, using 50 mM HEPES pH 8, 1 M NaCl, 2 mM TCEP, 5% glycerol. The nucleic acid-free TiLV-NP fractions (ratio A260/280 = 0.56) were dialyzed overnight at 4°C in a final buffer (50 mM HEPES pH 8, 300 mM NaCl, 0.5 mM TCEP, 5% glycerol), concentrated using Amicon Ultra (10 kDa cut-off), flash-frozen in liquid nitrogen, and stored at −80°C for further use.

### Size-exclusion chromatography with multi-angle light scattering

SEC-MALS experiments were performed on an OMNISEC system (Malvern) using a Superdex 200 Increase 10/300 GL column (Cytiva). The column was equilibrated with a buffer containing 50 mM HEPES pH 8, 150 mM NaCl, 2 mM TCEP. The system calibration was performed using a 50 μL injection of bovine serum albumin at 2 mg/ml to identify the monomeric and dimeric populations. Following calibration, 50 μL injections of apo TiLV-NP at concentrations of 5 and 3 mg/ml and in the presence of the 40-mer vRNA loop (5′-pGCA AAU CUU UCU CAC GUC CUG ACU UGU GAG UAA AAU UUG G-3′) (1 NP: 0.5 RNA molar ratio) at 3 mg/ml were performed. Static light scattering detection was conducted using the OMNISEC system's RALS and LALS detectors (Malvern) with a laser emitting at 660 nm. The weight-average molar masses were calculated using the OMNISEC v5.10 software (Malvern).

### Mass photometry analysis

Mass photometry measurements were performed on a OneMP mass photometer (Refeyn). Coverslips (No. 1.5H, 24  ×  50 mm, VWR) were washed with water and isopropanol before being used as a support for silicone gaskets (CultureWellTM 423 Reusable Gaskets, Grace Bio-labs). Contrast/mass calibration was realised using native marker (Native Marker unstained protein 426 standard, LC0725, Life Technologies) with a small field of view and monitored during 60 s using the AcquireMP software (Refeyn). For each condition, 18 μl of buffer (50 mM HEPES pH 8, 150 mM NaCl, 2 mM TCEP) were used to find the focus. Around 2 μl of sample were added to reach a final TiLV-NP concentration of 25 nM. Movies of 60 s were recorded, processed and mass estimation was determined automatically using the DiscoverMP software (Refeyn).

### Sample preparation, cryo-EM grid freezing and data collection

For sample preparation, 100 μM apo TiLV-NP were incubated with the 40-mer vRNA loop (5′-pGCA AAU CUU UCU CAC GUC CUG AUU UGU GAG UAA AAU UUG G -3′) (1:0.5 molar ratio) for 1h at 4°C in a SEC buffer containing 50 mM HEPES pH 8, 150 mM NaCl, 2 mM TCEP. The 40-mer vRNA loop was chosen based on preliminary cryo-EM data indicating that it could yield RNA-bound structures of TiLV-NP in diverse oligomeric states. Additionally, the potential presence of double-stranded regions might aid determination of RNA directionality. The resulting mixture was centrifuged 5 min at 11 000 g prior to injection onto a Superdex 200 Increase 3.2/300 column (Cytiva). To enrich for larger RNA-bound oligomers (≥ tetramers), two adjacent left-side fractions of the main SEC peak were collected and used to optimise the TiLV-NP-RNA concentration used for cryo-EM grid preparation. For cryo-EM grid preparation, 1.5 μl of sample was applied on each side of plasma cleaned (Fischione 1070 Plasma Cleaner: 1 min 30, 80% oxygen, 20% argon) grids (UltrAufoil 1.2/1.3, 300 mesh). Excess solution was blotted for 3 s, blot force −2, at 100% humidity and 4°C with a Vitrobot Mark IV (ThermoFisher) before plunge freezing in liquid ethane.

Two automated data collections were performed on two different grids using a TEM Krios (ThermoFisher) operated at 300 kV equipped with a K3 direct electron detector camera (Gatan) using SerialEM [25]. Coma and astigmatism correction were performed on a carbon grid. Movies of 40 frames were recorded in counting mode at a × 105 000 magnification, giving a pixel size of 0.822 Å, with defocus ranging from −0.8 to −2.0 μm. Total exposure dose was ∼40 or ∼50 e^−^/Å^2^.

### Image processing

For the two collected datasets, the image processing followed a similar workflow carried out in CryoSPARC v4.3 and v4.5 [26]. Movie drift correction was performed using all frames, along with gain reference and camera defect corrections. CTF parameters were determined using ‘Patch CTF estimation’. Realigned micrographs were then inspected and low-quality images were manually discarded. RNA bound TiLV-NP oligomers were automatically picked using a circular blob with diameters ranging from 80 to 140 Å. Particles were extracted, 2D classified, and subjected to an ‘Ab-initio reconstruction’ job to generate multiple initial 3D reconstructions. The best 3D models were used for one round of heterogeneous refinement and the resulting 3D classes subjected to a non-uniform 3D refinement job. The resulting maps were used to prepare 2D templates and particles picked using the template picker job. Resulting particles were combined, with duplicates removed, and extracted using a box size of 340  ×  340 pixels^2^. Successive 2D classifications were used to eliminate particles displaying poor structural features. A second heterogeneous refinement was performed using tetramers (pseudo-C2 and pseudo-C4), pentamer (pseudo-C5), and hexamer (pseudo-C6) maps as initial 3D models. Particles belonging to a specific oligomer were re-centered and re-extracted, 2D classified to ensure that there is the least misclassification, and subjected to non-uniform 3D refinement, which was then used for reference-based motion correction [27]. Motion-corrected particles were subjected to a last non-uniform 3D refinement and resulted in four final maps, one for each RNA-bound TiLV-NP oligomers (pseudo-C2/-C4/-C5/-C6).

The inherent flexibility between each TiLV-NP prevented application of a defined symmetry. Based on the overall complete C1 maps (pseudo-C2/-C4/-C5/-C6), symmetry expansion was performed using C2/C4/C5 and C6 transformation, respectively. To better account for NP–NP interactions, two TiLV-NPs were kept while the remaining regions were subtracted. The resulting particles were used for local 3D refinement. Finally, 3D classification without alignments were performed to isolate the most homogeneous subset of particles, itself subjected to a final local refinement, resulting in four final maps enclosing 2 TiLV-NPs for each RNA-bound TiLV-NP oligomers (pseudo-C2/-C4/-C5/-C6).

Post-processing was performed in CryoSPARC using an automatically determined B-factor. For each final map, reported global resolution is based on the FSC 0.143 cut-off criteria. Local resolution variations were estimated in CryoSPARC.

For detailed image processing information, please refer to Supplementary Figs S1 and S2. For assessing cryo-EM map quality, please refer to Supplementary Figs S3, S4, and S8.

### Model building and refinement

Model building of the nearly full-length TiLV-NP (lacking the first 33 residues) and associated RNA (deposited with generic purine or pyrimidine bases) was performed *de novo* in COOT [28] using the map corresponding to two RNA-bound NPs extracted from the TiLV-NP pseudo-C5 pentamer, at 2.9 Å resolution. Models were refined using Phenix real-space refinement [29] with Ramachandran restraints. The TiLV-NP model was duplicated and rigidly fitted into the density for each NP within each oligomer. The oligomerisation loop and hinges, the RNA and other flexible loops were then manually adjusted into the cryo-EM density, followed by one round of refinement. For the RNA, the exact sequence register could not be determined and purines or pyrimidines were assigned depending on apparent base size, whilst maintaining Watson–Crick base-pairing in the double-stranded regions. In the deposited PDBs, generic purine (P5P) or pyrimidine (Y5P) nucleotides were substituted. The CC_mask_ is relatively low for the pseudo-C4 and pseudo-C6 structures, attributable to poorer density for 2 and 3 NPs, respectively. Atomic model validation (Supplementary Tables S1–S4) was performed using MolProbity [30] as implemented in Phenix. Model resolution according to the cryo-EM map was estimated at the 0.5 FSC cutoff. Electrostatic potential was calculated using PDB2PQR and APBS [31]. Figures were generated using ChimeraX [32].

## Results

### Expression, purification and biophysical characterisation of apo TiLV-NP and RNA-bound TiLV-NP oligomers

**Figure 1.**
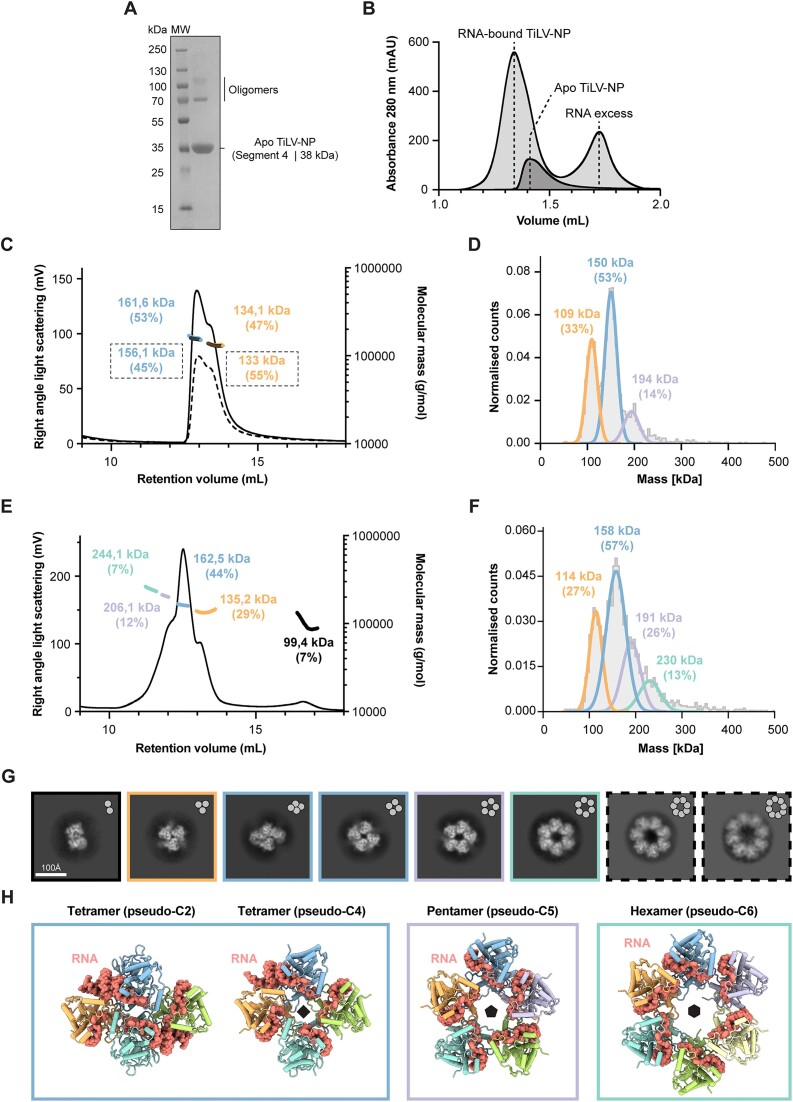
Biochemical, biophysical, and structural characterisation of TiLV-NP. (**A**) SDS-PAGE analysis of purified apo TiLV-NP. The molecular ladder (MW) is shown on the left side of the gel. Apo TiLV-NP is indicated on the right side of the gel (segment 4; 38.4 kDa). Despite denaturing conditions, apo TiLV-NP oligomers remain visible and indicated on the right side of the gel. (**B**) SEC profiles of RNA-bound TiLV-NP (light grey) and apo TiLV-NP (dark grey). The relative absorbance at 280 nm (mAU) is on the *y*-axis. The elution volume (mL) is on the *x*-axis, graduated every 100 μL. (**C**) SEC-MALS of apo TiLV-NP injected at 5 mg/mL (black solid line) and 3 mg/mL (black dotted line). The right angle light scattering (mV) is on the left *y*-axis, the molecular mass (g/mol) on the right *y*-axis. The elution volume (mL) is on the *x*-axis, graduated every 1 mL. The molecular masses of apo TiLV-NP tetramers and trimers are coloured in blue and orange, outlined for the 3 mg/mL run. (**D**) Mass photometry analysis of apo TiLV-NP. The curves and molecular masses for apo TiLV-NP pentamers, tetramers and trimers are coloured mauve, blue and orange, respectively. (**E**) SEC coupled with MALS of RNA-bound TiLV-NP injected at 3 mg/mL (black solid line). The right-angle light scattering (mV) is on the left *y*-axis, the molecular mass (g/mol) on the right *y*-axis. The elution volume (mL) is on the *x*-axis, graduated every 1 mL. The molecular masses determined for RNA-bound TiLV-NP hexamers, pentamers, tetramers, trimers, and dimers are, respectively, coloured green, mauve, blue, orange, and black. (**F**) Mass photometry analysis of RNA-bound TiLV-NP. The curves and molecular masses for apo TiLV-NP hexamers, pentamers, tetramers, and trimers are coloured green, mauve, blue, and orange, respectively. TiLV-NP dimers are not detected. (**G**) Cryo-EM 2D class averages of RNA-bound TiLV-NP oligomers. Dimers and trimers are outlined in black and orange, respectively. Tetramers are present in two distinct conformations and outlined in blue. Pentamers and hexamers are outlined in mauve and green, respectively. Heptamers and octamers, which are not detected in SEC-MALS or mass photometry, are outlined with black dotted lines. (**H**) Cartoon representation of the different RNA-bound TiLV-NP structures solved by cryo-EM. Each TiLV-NP is coloured differently (orange, blue, mauve, yellow, green, and cyan). The RNA is displayed as spheres, coloured in salmon..

TiLV-NP is a 38.4 kDa protein composed of 354 amino acids and encoded on the TiLV segment 4 [24]. TiLV-NP was expressed in insect cells and purified in its apo form, as confirmed by an A260/280 ratio of 0.56 indicating the absence of nucleic acids (Fig. 1A and B; see ‘Material and methods’). Standard size exclusion chromatography (SEC) revealed that apo TiLV-NP elutes as a broad peak, suggesting the presence of multiple oligomeric states in solution, as seen for instance for FluA/NP [13]. Further analysis using SEC coupled with multi-angle light scattering (SEC-MALS) showed that apo TiLV-NP predominantly forms trimers and tetramers in roughly equal proportions, regardless of protein concentration. At 5 mg/ml, ∼53% tetramers and ∼46% of trimers were detected, while at 3 mg/ml, it formed ∼45% tetramers and ∼54% trimers (Fig. 1C). The experimental molecular mass of the apo TiLV-NP tetramers (5 mg/ml: 161.6 kDa; 3 mg/ml: 156.1 kDa) is within 5% of the theoretical value (153.6 kDa). However, the apo TiLV-NP trimeric form showed a larger discrepancy with the theoretical molecular mass (115.2 kDa) corresponding to only 86% of the experimental value (134.1 and 133 kDa), perhaps indicating some flexibility or conformational changes (Fig. 1C). Complementary mass photometry analysis, performed at nM protein concentration (versus μM for SEC), yielded similar results. Two main peaks are observed at 109 kDa (trimers, 33%) and 150 kDa (tetramers, 53%). In addition, a third less-resolved peak, corresponding to a pentameric form was detected in low amounts (14%), but not detected in SEC-MALS (Fig. 1D).

To reconstitute RNA bound TiLV-NP oligomers, apo TiLV-NP was incubated with the previously described 40-mer vRNA loop (vRNA loop, 5′-pGCA AAU CUU UCU CAC GUC CUG AUU UGU GAG UAA AAU UUG G -3′), which corresponds to the joining of the partially complementary first (5′) and last (3′) 20 nucleotides of the ends of TiLV vRNA segment 9 [4]. Comparative SEC analysis indicated a shift and peak broadening in the presence of RNA compared to the apo TiLV-NP, suggesting an enhanced oligomerisation (Fig. 1B). This was further confirmed by SEC-MALS, which showed that RNA-bound TiLV-NP forms oligomers ranging from dimers to hexamers (Fig. 1E). Trimers and tetramers were still the most abundant, as for apo TiLV-NP, with ∼44% and ∼29% of the total population, respectively. The pentameric form accounted for ∼12%, while the hexameric and dimeric forms are present at ∼7% each. The experimental molecular masses matched closely with the theoretical ones if it is assumed that TiLV-NP oligomers are bound to at least one copy of the vRNA loop (12.8 kDa): 244.1 kDa (experimental) versus 243.2 (theoretical) for hexamers (99.6%), 206.1 kDa versus 204.8 for pentamers (99.4%), 162.5 kDa versus 166.4 for tetramers (102.4%), 135.2 kDa for 128 for trimers (94.6%), and 99.4 kDa for 89.6 for dimers (90.1%) (Fig. 1E). The presence of dimers, not detected in the apo TiLV-NP SEC-MALS analysis, suggests that apo TiLV-NP trimers and tetramers could possibly dissociate to accommodate RNA binding and subsequently form higher-order oligomers. Complimentary mass photometry analysis validated these findings with an increased presence of pentamers (26%) and the appearance of hexamers (13%), although peak resolution remains limited. The dimeric population is not observed (Fig. 1F).

Taken together, these biophysical results show that apo TiLV-NP exist predominantly in trimeric and tetrameric forms, whilst RNA binding drives the oligomerisation of TiLV-NP towards higher order oligomers, principally pentamers, and hexamers.

### Cryo-EM structure of RNA-bound TiLV-NP and comparison with the influenza A/NP

To structurally characterise RNA-bound TiLV-NP oligomers, a mixture of vRNA loop and TiLV-NP was incubated and loaded on SEC (see ‘Materials and methods’). To enrich for high-order oligomers (≥pentamers), different early fractions of the SEC peak were deposited on grids, vitrified, and imaged by cryo-EM (Supplementary Figs S1 and S2). Initial 2D classifications revealed a diverse population of RNA-bound TiLV-NP oligomers, with pseudo-symmetry from C2 to C8, with octamers being the highest observed oligomeric state (Fig. 1G). No pseudo-helical assemblies were detected under these *in vitro* conditions.

Following cryo-EM data collection and image processing, multiple pseudo-symmetrical TiLV-NP oligomer structures bound to the vRNA loop were resolved, with overall resolution ranging from 2.9 to 3.7 Å (Fig. 1H; Supplementary Tables S1–S4; Supplementary Figs S1–S3). These structures include two distinct tetrameric forms (pseudo-C2 and pseudo-C4), one pentameric (pseudo-C5), and one hexameric (pseudo-C6) form. The pseudo-C2 and pseudo-C5 oligomers appeared the most stable with uniform density according to local resolution, while the pseudo-C4 and pseudo-C6 structures displayed poorer density for 2 and 3 NPs, respectively (Supplementary Figs S1–S3). To account for this variability, symmetry expansion, particle subtraction, 3D classification without alignments, and local refinements were applied during cryo-EM image processing (see ‘Materials and methods’). This enabled isolation and refinement of the most homogeneous sub-oligomers, corresponding to two NPs within each oligomer. This strategy improved map quality allowing unambiguous assignment of the TiLV-NP sequence, analysis of NP–NP interactions and determination of the RNA polarity (Supplementary Figs S1–S4).

Structure solution of higher oligomeric states (≥pseudo-C7) was hindered by the low number of particles and absence of side views. Conversely, lower oligomeric state structures (≤pseudo-C3) lacked sufficient signal-to-noise ratio, primarily due to the ice thickness relative to the particle size, impeding accurate particle picking, sorting, and angle estimation for 3D reconstruction.

**Figure 2.**
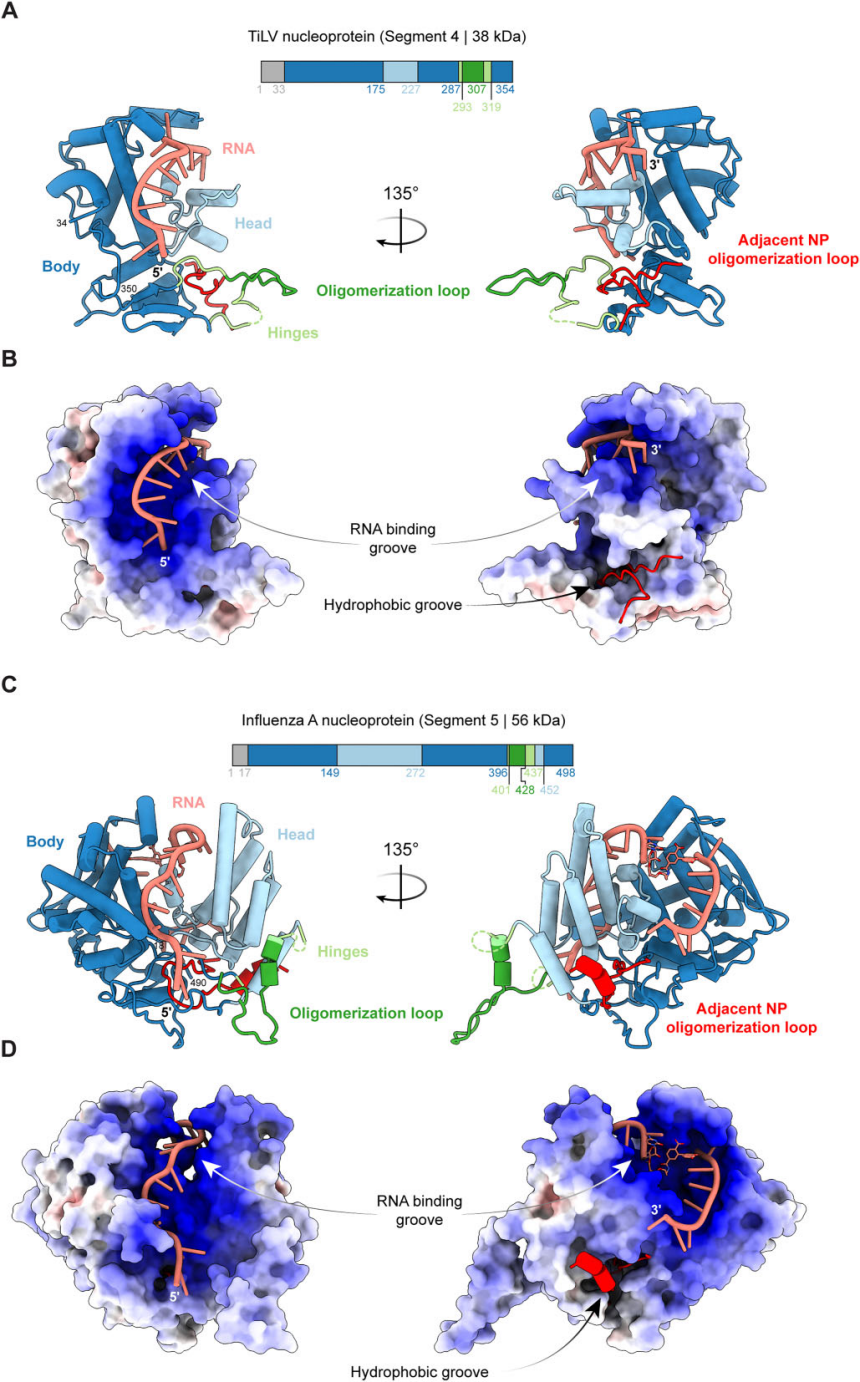
Comparison of the structure of RNA-bound TiLV-NP with that of FluA/NP. (**A**) Schematic of the TiLV-NP domains and cartoon representation of the RNA-bound TiLV-NP structure. The first N-terminal 33 residues are unseen (grey). The body domain is coloured in dark blue, the head domain in light blue, the hinges in light green, the RNA in salmon, and the oligomerisation loop in dark green. The adjacent NP oligomerisation loop is coloured in red. (**B**) Electrostatic surface representation of TiLV-NP structure. Positively charged regions (+10) are dark blue, white is neutral (0) and red is negative (-10). The RNA binding groove and the hydrophobic groove, in which the adjacent NP oligomerisation loop docks, are annotated. (**C**) Schematic of the FluA/NP domains and cartoon representation of the RNA-bound FluA/H1N1-NP structure (PDB 9GAT). The first N-terminal 17 residues are unseen (grey). All domains are coloured as for TiLV-NP in panel A. (**D**) Electrostatic surface representation of FluA/H1N1-NP structure (PDB 9GAT) structure, coloured and annotated as in B.

TiLV-NP is 30% smaller than the influenza A nucleoprotein (FluA/NP), consisting of 354 amino acids versus 498 for FluA/NP. This size reduction is similar to that observed for the heterotrimeric TiLV viral polymerase, which is only 60% the size of the influenza A polymerase [4]. Nevertheless, TiLV-NP adopts a fold, rich in α-helices, which is topologically similar to FluA/NP. Like orthomyxovirus NPs, TiLV-NP has a crescent shape divided into three subdomains (Fig. 2; Supplementary Fig. S5). Of the 354 residues, the first 33 N-terminal amino acids are not visible in any of the cryo-EM maps, presumably due to flexibility. The TiLV-NP ‘body’ domain comprises residues 34–175, 228–287, 320–350, and contains an extended oligomerisation loop (294–307) flanked by flexible hinges (288–293; 308–319). The ‘head’ domain (176–227) is inserted within the body domain and separates two critical regions (Fig. 2A, Supplementary Fig. S5A). On one side, along with the body domain, is formed a positively charged RNA-binding groove that can accommodate up to 12 nucleotides (Fig. 2A and B) and on the other side, a hydrophobic groove into which the oligomerisation loop (‘tail-loop’ in Flu/NP) of an adjacent TiLV-NP can insert (Fig. 2B). Compared to FluA/NP (Fig. 2C and D), the TiLV-NP body domain is the least reduced, retaining 80% of its size (230 versus 287 residues). In contrast, the TiLV-NP head domain is the most minimised, consisting of far fewer α-helices and being only 38% the size of the FluA/NP counterpart (51 residues versus 136) (Supplementary Fig. S5). Finally, the TiLV-NP oligomerisation loop and both hinges are also reduced in size and do not project as far as in FluA/NP (31 versus 40 residues) (compare Fig. 2A–D).

### Overall structure of the RNA-bound TiLV-NP oligomers and NP–NP interactions

**Figure 3.**
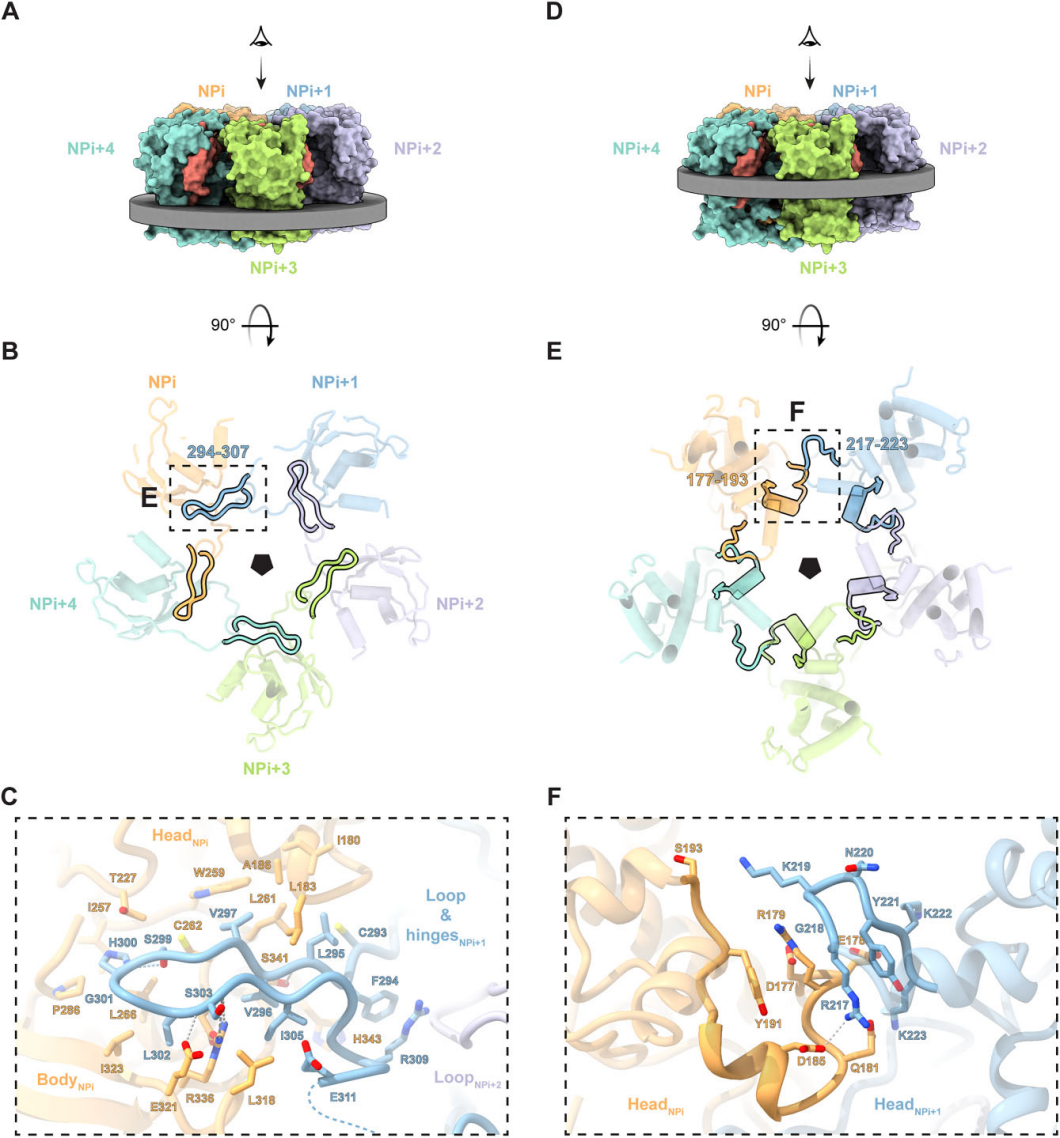
TiLV-NP oligomerisation and NP–NP interfaces. (**A**) Surface representation of the TiLV-NP pseudo-C5 structure observed perpendicular to the pseudo-C5 symmetrical axis. NPs are annotated and coloured orange (NPi), dark blue (NPi + 1), mauve (NPi + 2), green (NPi + 3), and cyan (NPi + 4). The section shown in panel B is indicated by a grey disk. (**B**) Cut-away view along the pseudo-C5 symmetric axis focusing on the oligomerisation loop interactions. Each NP is annotated and coloured as in panel A. The oligomerisation loop (294–307) of each NP is outlined by black solid lines. The dotted square indicates the region presented in panel C. (**C**) Close-up view of the interaction between the TiLV-NPi + 1 oligomerisation loop and the TiLV-NPi body and head domains. NPs are coloured as in B. Interacting residues are shown as non-transparent sticks. Ionic and hydrogen bonds are shown as grey dotted lines. (**D**) As A, but with a different grey disk corresponding to the section shown in E. (**E**) Cut-away view along the pseudo-C5 symmetric axis focusing on the head-to-head interaction. Each NP is annotated and coloured as in panel D. The head-to-head interactions (residues 177–193 and 217–223) are highlighted with black solid lines. The dotted square indicates the region presented in panel F. (**F**) Close-up view of the interaction between the TiLV-NPi head (177–193) and TiLV-NPi + 1 head (217–223) domains. NPs are coloured as in panel E. Interacting residues are shown as non-transparent sticks. Ionic and hydrogen bonds are shown as grey dotted lines.

The RNA-bound TiLV-NP structure has been determined as part of distinct pseudo-symmetrical oligomers, including two tetrameric forms with pseudo-C2 and pseudo-C4 symmetries (Fig. 1H). The pseudo-C2 tetramer adopts an elongated structure, measuring 116 Å in length, 99 Å in width, and 70 Å in height (Supplementary Fig. S6A). In contrast, the pseudo-C4 tetramer is more compact, with dimensions of 95 Å in length, 102 Å in width, and 62 Å in height (Fig. 1H; Supplementary Fig. S6B). This structural variability is due to distinct RNA binding modes at the NP–NP interfaces. Within the pseudo-C2 tetramer, two A-form RNA double-stranded helices are inserted at the interfaces between opposite pairs of NP, whose single-stranded 5′ and 3′ extensions each bind one of the four NPs (Fig. 1H; Supplementary Fig. S6A). For the pseudo-C4 tetramer, the observed RNA is mostly single stranded, with one less well-ordered, double-stranded region (Fig. 1H; Supplementary Fig. S6B). Consequently, the NP-NP interface areas differ. The pseudo-C2 tetramer exhibits mean interface area of 1294 Å^2^ between NPs within one dimer and 994 Å^2^ at the dimer interface, while the pseudo-C4 tetramer exhibits a more uniform interface of 1313 Å^2^ between NPs. In addition to the two tetramers, pentameric and hexameric oligomers were also resolved, displaying pseudo-C5 and pseudo-C6 symmetries (Fig. 1H; Supplementary Fig. S6C and D). These higher-order oligomers resemble the pseudo-C4 structure but incorporate additional NPs, resulting in circular assemblies with diameters of ∼107 Å for the pentamer and ∼122 Å for the hexamer (Supplementary Fig. S6C and D). The NP–NP interface areas are smaller, averaging 1240 Å² for the pentamer and 1065 Å² for the hexamer. The accommodation of an A-form double-stranded RNA helix at the pseudo-C2 NP interface, as well as the oligomerisation into circular pentamers and hexamers, is facilitated by the flexible hinges of TiLV-NP (residues 288–293, 308–319). These allow for slight shifts and rotations between adjacent NPs, whilst maintaining strong NP–NP interactions via the interpenetrating oligomerisation loop (Fig. 3A–C; Supplementary Fig. S6A–D).

TiLV-NP oligomerisation is mediated by two main contact areas between adjacent NPs (Fig. 3A–C). The primary interaction, conserved for all oligomers, is formed by the β-hairpin-like oligomerisation loop of TiLV-NPi + 1 (residues 293–308 and E311), which inserts into a hydrophobic pocket formed by both the body (residues 336–343, 318–324, 257–266, 286–289) and head (residues 180–186, 227) domains of the adjacent TiLV-NPi (Fig. 3A–C). Key interactions include the stacking of TiLV-NPi + 1 F294, stabilised by R309, with TiLV-NPi H343. The first strand of TiLV-NPi + 1 293-CFLVVA is stabilised by TiLV-NPi head (I180, L183, A185 residues) and TiLV-NPi + 1 body (L261, W259 and C262 residues) (Fig. 3C). TiLV-NPi + 1 tip residues S299 and H300 are inserted into a pocket formed by TiLV-NPi T227, I257 and L266, with TiLV-NPi + 1 G301 stabilised by TiLV-NPi I323 (Fig. 3C). The second strand (TiLV-NPi + 1 302-LSAI) is stabilised by TiLV-NPi E321, R336, L318, with TiLV-NPi + 1 L302 being clamped between TiLV-NPi I323, E321, and R336 (Fig. 3C). TiLV-NPi + 1 S303 amine and carboxyl groups are hydrogen-bonded to TiLV-NPi E321 and R336, respectively (Fig. 3C). These interactions account for ∼82% (∼940A^2^) of the total interface area between adjacent TiLV-NPi + 1 and TiLV-NPi, making them essential for oligomeric assembly.

**Figure 4.**
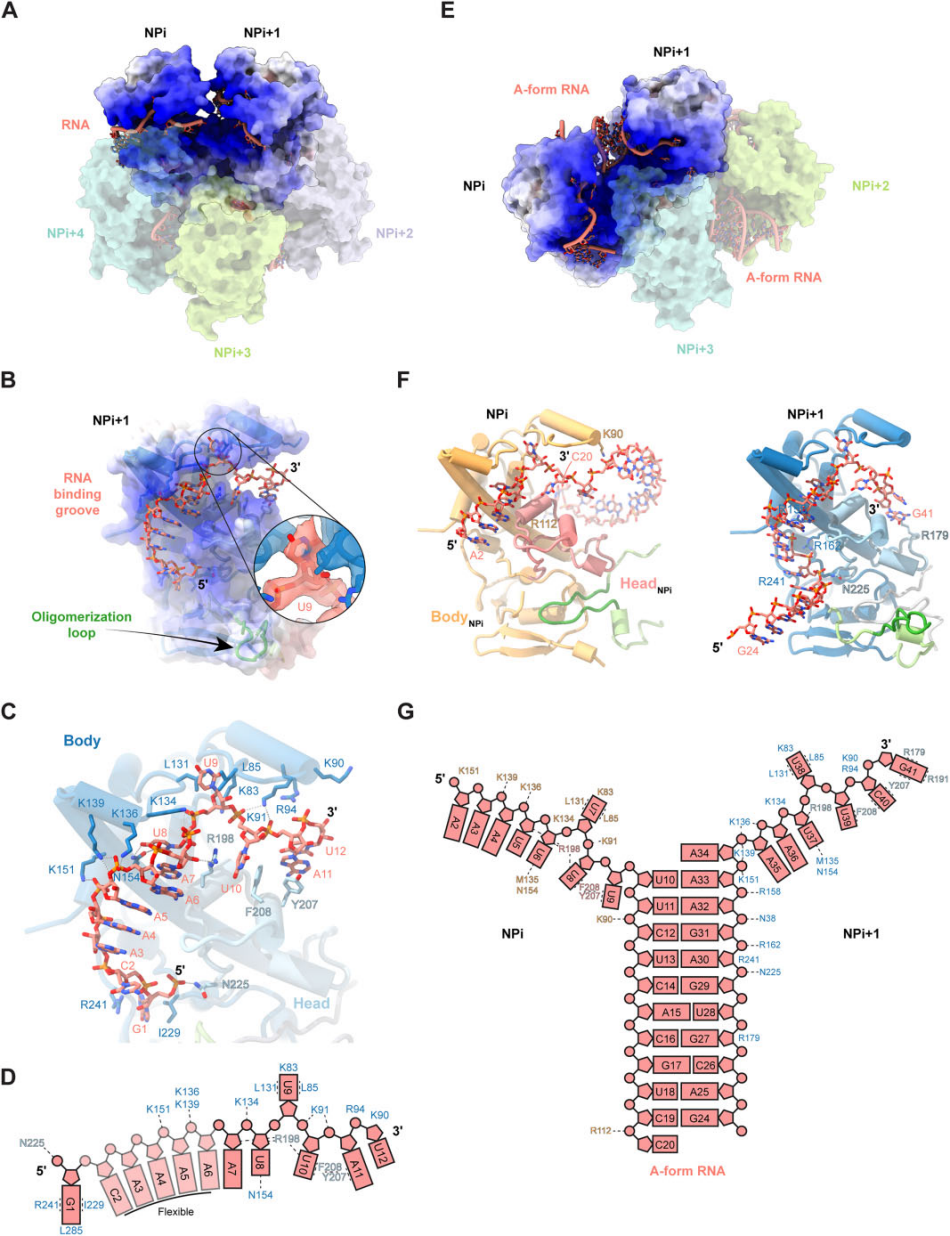
RNA structure, interactions, and polarity within the TiLV-NP. (**A**) Surface representation of the RNA-bound TiLV-NP pseudo-C5 structure. Two NPs (NPi, NPi + 1) are shown with electrostatic surface representation where dark blue represents positively charged regions (+20), white is neutral (0), and red is negative (-20). NPi + 2, NPi + 3 and NPi + 4 are coloured as in Fig. 1A, and shown transparent. The RNA, coloured in salmon, binds as a single strand within each NP. (**B**) Close-up view of the RNA binding groove in TiLV-NPi + 1 from the TiLV-NP pseudo-C5 structure. The electrostatic surface is shown transparent with the cartoon representation of the TiLV-NP structure underneath. The RNA is shown as sticks. The 5′ and 3′ ends are indicated. The 5′ end follows the oligomerisation loop orientation. A close-up view of the Coulomb potential around U9 unambiguously reveals the RNA polarity. (**C**) Close-up view of the interaction between the RNA and the TiLV-NP body and head domains. TiLV-NP domains are coloured as in Fig. 2A. Interacting residues are shown as non-transparent sticks. Ionic and hydrogen bonds are shown as grey dotted lines. The extracted RNA density from the local refinement around 2 NPs from the TiLV-NP pseudo-C5 cryo-EM map (2.9 Å resolution) unambiguously show the RNA polarity. (**D**) Schematic summary of the interactions between TiLV-NPi + 1 and the vRNA loop (from the local refinement of 2 NPs from the pseudo-C5 structure). The RNA is coloured salmon. Interacting residues are coloured based on the domain they belong to, dark blue for the body domain and light blue for the head domain. Flexible nucleotides are transparent and indicated. (**E**) Surface representation of the RNA-bound TiLV-NP pseudo-C2 structure. Two NPs (NPi, NPi + 1) are shown with the electrostatic surface representation, where dark blue is positively charged (+20), white is neutral (0) and red is negative (-20). NPi + 2 and NPi + 3 are coloured as in Fig. 1A, shown transparent. The RNA binds as a single strand within each NP, forming an A-form double-stranded RNA helix at the interface of NPi/NPi + 1 and NPi + 2/NPi + 3. (**F**) Cartoon representation of TiLV-NPi and TiLV-NPi + 1 from the TiLV-NP pseudo-C2 structure. Only the RNA strand interacting with each respective NP is shown for clarity. Interacting residues not observed in the TiLV-NP pseudo-C5 structure are indicated. TiLV-NPi body and head domains are coloured orange and salmon, respectively. TiLV-NPi + 1 domains are coloured as in Fig. 2A. (**G**) Schematic summary of the interactions between TiLV-NPi, TiLV-NPi + 1 from the local refinement around 2 NPs (pseudo-C2 structure) and the vRNA loop. The RNA is coloured salmon. Interacting residues are coloured according to the domain they belong as in panel F.

The second area is located at the interface between both head domains of TiLV-NPi (177–193) and TiLV-NPi + 1 (217–223) (Fig. 3D–F). Notably, TiLV-NPi D185 interacts with TiLV-NPi + 1 R217, while TiLV-NPi Y191 stacks with R179, which in turn interact with TiLV-NPi + 1 G218 (Fig. 3F). Additionally, TiLV-NPi S193 is close to TiLV-NPi + 1 K219, and TiLV-NPi E178 is surrounded by TiLV-NPi + 1 residues 220–223. This secondary interaction is less consistent between NPs, suggesting accommodation depending on the NPi-NPi + 1 angle. Conversely, positively charged residues within the 211–225 loop of the head domain are also implicated in the formation of the TiLV-NP RNA binding groove and RNA stabilisation, described in more detail below (Fig. 4).

### TiLV-NP RNA binding mode

Within each TiLV-NP oligomer, all NPs bind the RNA as a single strand within a positively charged binding groove formed by both the body and head domains. The RNA is directed towards the central part of each oligomer and is thus protected from solvent, as also observed in NP oligomers from bunyaviruses (Fig. 4A and B; Supplementary Fig. S7). In contrast, the corresponding positively charged groove is solvent exposed in the trimeric or tetrameric apo- FluA/H5N1-NP, FluA/H1N1-NP, FluB/Managua/2008-NP, and FluD/Bovine/France-NP oligomers, previously characterised using X-ray crystallography (Supplementary Fig. S7).

In the pseudo-C5 oligomers, up to 12 nucleotides can be modelled per NP (Fig. 4A–D). The RNA-binding mode is conserved across all NPs, with ∼930 Å^2^ of RNA surface buried within each groove, making the RNA largely inaccessible to the solvent. Only discontinuous RNA density is observed at the NP–NP interfaces suggesting two potential binding modes: either individual vRNA loops interact separately with each NP, or a single vRNA loop may bridge two adjacent NPs, leaving a flexible RNA loop at the NP-NP interface.

In contrast, the pseudo-C2 oligomer structure exhibits a distinct RNA-binding mode (Fig. 4E). Within each of the positively charged grooves of TiLV-NPi and TiLV-NPi + 1, 8 nucleotides of RNA are bound as in the pseudo-C5 oligomers, but with free 5′ and 3′ ends, respectively (Fig. 4F). These two strands then come together at the NP-NP interface to form an A-form RNA double-helix comprising at least 10 base-pairs with a total of around 37 nucleotides visible (Fig. 4G).

Two such structures bind to the pseudo-C2 oligomer (Fig. 1H). Due to the inability to precisely define bases, the exact nucleotide register remains elusive and in the modelling, purines or pyrimidines were assigned depending on apparent base size, whilst maintaining Watson-Crick base-pairing in the double-stranded regions. However, since the 40-mer vRNA loop used in the reconstitutions corresponds to 20 nucleotides from the 3′ and 5′ ends of one genome segment, the two observed RNA structures could each correspond to a single vRNA loop with a promoter-like fold, i.e. with a distal duplex (with the loop density missing), and single stranded terminii splayed out to bind in the two neighbouring NPs. Less likely, each RNA moiety could result from partial hybridisation of two extended vRNA copies.

Importantly, both the quality of the RNA density in the 2.9 Å resolution focused map of covering two adjacent NPs from the pseudo-C5 oligomer and the A-from double-stranded RNA helix in the pseudo-C2 oligomer unambiguously reveal the 5′ to 3′ RNA directionality of the RNA bound to TiLV-NP. Moreover, this directionality is consistent between all modelled NPs in all oligomers where the RNA density is sufficiently well-resolved (Fig. 4, Supplementary Fig. S8).

RNA binding to TiLV-NP is primarily mediated by (i) interactions between the phosphate backbone and multiple positively charged residues, (ii) base stacking, and (iii) key interactions with the O2′ hydroxyl group, enabling TiLV-NP to selectively bind RNA over DNA (Fig. 4C and D). As mentioned, in the 2.9 Å resolution structure derived from the focused map of two NPs from the pseudo-C5 oligomer, a total of 12 nucleotides are modelled within one NP (Fig 4C and D). Starting from the 5′ to the 3′ end, the G1 phosphate is stabilised by N225 from the head domain, with its base nestled in a pocket formed by I229, R241, and L285 of the body domain. Weaker density is observed from C2 to A4 as they are not directly stabilised by TiLV-NP but rather stack with each other until reaching U8. A5 and A6 phosphates interact with a triplet of positively charged residues K151, K136, and K139, while A7 ribose O2′ is stabilised by hydrogen bonding with R198 from the head domain. The phosphate group of U8 interacts with K134, and its base is stabilised by N154. U9 takes a sharp turn, repelled by R198, with its base sandwiched between L131, L85 and K83. U10 and A11 phosphates are stabilised by K91, while U10 base is wedged between R198 and F208, which stacks with Y207 to further stabilise A11 base. Finally, U12 phosphate group is surrounded by positively charged residues (R94, K90), while its base stacks with A11 (Fig. 4C and D). Following U12, only disjointed density is observed precluding further RNA model building.

In the pseudo-C2 oligomer structure, continuous density is observed for 37 nucleotides. Within the RNA binding grooves of TiLV-NPi and TiLV-NPi + 1, 8 (A2-U9 and A34-G41) nucleotides are stabilised in a similar manner to the pseudo-C5 oligomer (Fig. 4D). However, at the interface of two NPs, the A-form RNA double helix (U10-A33) is formed with the complementary strand extending into the adjacent TiLV-NPi + 1 (Fig. 4F and G). In addition to the Watson–Crick base pairing, additional polar residues stabilise the RNA phosphate backbone. Of note, within TiLV-NPi, C20 phosphate is stabilised by R112. In TiLV-NPi + 1, R179 and R241 residues stabilise G27 and A30 ribose, with N225, R162, N38, and R158, respectively, stabilising the 5′ phosphates of A30, G31, A32, and A33. A34 stacks on U10 and A33 bases with its phosphate being stabilised by K151, K139 (Fig. 4F and G).

Supporting our structural findings, a previous study used a yeast three-hybrid system to mutate specific positively charged residues of TiLV-NP and assess their impact on RNA binding [24]. Mutants ‘K90A-K91A-R92A-R94A’ and K134A significantly disrupted the interaction of TiLV-NP with the RNA. This effect is consistent with our structures, which show that residues K90, K91, R94 and K134 interact with the RNA phosphate backbone, whereas R92 is solvent-exposed and does not interact with the RNA (Fig. 4C). Conversely, substitutions K136A and R158A did not significantly impair RNA binding [24], which is in agreement with our RNA-bound TiLV-NP structures. K136, though interacting with the phosphate RNA backbone, is surrounded by multiple positively charged residues (K134, K139) that may compensate for its alanine substitution. Similarly, R158, which interacts with the A33 phosphate group in the pseudo-C2 tetramer as part of the A-form RNA double helix, does not interact with RNA in other oligomers. This residue thus likely contributes to the positively charged RNA-binding groove and stabilises the NP fold, rather than being critical for direct RNA interaction (Fig. 4G).

## Discussion

Numerous X-ray structures of orthomyxoviral NPs, such as influenza A [12,14,15], B [16], D [17], THOV [18], and ISAV [19], have been determined as small oligomers without bound RNA (Supplementary Fig. S5). The only RNA-bound X-ray structure is that of FluA/H5N1-NP with three 2′OH methylated nucleotides bound to a basic pocket [15] (Supplementary Fig. S9). Structural studies using cryo-electron microscopy/tomography on purified viral RNPs revealed the overall structures of native FluA [7, 20] and THOV [18] RNPs, at nanometer resolution, too low to visualise the RNA. These studies report anti-parallel left-handed assemblies with helical rises of 28 and 24.6 Å, and helical twists of −57° to −64° and −55.5°, for Flu and THOV, respectively. In contrast, an atomic force microscopy study showed a right-handed RNP structure [22]. More recently, a cryo-EM study at ∼6 Å resolution of FluA/H1N1-NP reconstituted with a 12-mer RNA oligomer labelled at the 3′ end with 6-fluorescein amidite (FAM), revealed a right-handed, super-helical RNP-like structure, but with parallel rather than the expected anti-parallel strands. Low-resolution density for 10–12 nucleotides was located at the NP-NP interface, but with unspecified directionality [21] (Supplementary Fig. S9). In addition, several functional studies have identified critical residues in FluA/NP responsible for oligomerisation and RNA binding [12] and attempts to model the recruitment of NPs into RNPs have been made [33]. However, apart from a newly published study [23], discussed in more detail below, no prior published work has provided insights into how RNA binds within the positively charged groove of NP, nor into the RNA 5′ to 3′ directionality, which is crucial for understanding genome encapsidation during the articulaviral replication process.

Here we present the biophysical and structural characterisation of RNA-bound oligomers of NP from TiLV, the prototypical Amnoonvirus. We show that, *in vitro*, apo TiLV-NP primarily forms tetramers and trimers, and in the presence of RNA, oligomerises into higher-order closed, planar oligomers, including pentamers, hexamers and larger assemblies, with each NP bound to RNA (Fig. 1). The TiLV-NP sequence is only similar to putative NPs from other Amnoonviruses. The most closely related is the recently reported Fancy Tailed Guppy fish virus segment 4 protein with 90% homology [34] (Supplementary Fig. S10). Much more distant sequences from Flavolineata, Namensis and Asotus viruses [35] show the most conservation between residues 230 and 305, comprising part of the body domain, the hinges and oligomerisation loop (Supplementary Fig. S10). Despite the complete divergence of sequence and having a significantly reduced size compared to classical orthomyxovirus NPs, TiLV-NP maintains the same fold and architecture (Fig. 2). It comprises body and head domains, along with a protruding oligomerisation loop flanked by flexible hinges. These conserved structural elements form a positively charged RNA binding groove and a hydrophobic pocket that binds a neighbouring oligomerisation loop thus mediating NP assembly formation (Fig. 3). In addition, we describe how TiLV-NP binds RNA and reveal the 5′ to 3′ RNA directionality (Fig. 4). This is unambiguously supported by both the high-resolution of focused cryo-EM maps and the presence of an A-form RNA double helix at the NP–NP interface of the pseudo-C2 oligomer. This observation highlights that inter-NP flexibility allows accommodation of RNA secondary structures between the NPs. Secondary structures have been detected in influenza A vRNA [36–38], as well as the irregular positioning of NP along the RNP [39]. These phenomena have been postulated to mediate intersegment RNA–RNA interactions and be implicated in selective bundling of influenza vRNPs prior to packaging in budding virions.

**Figure 5.**
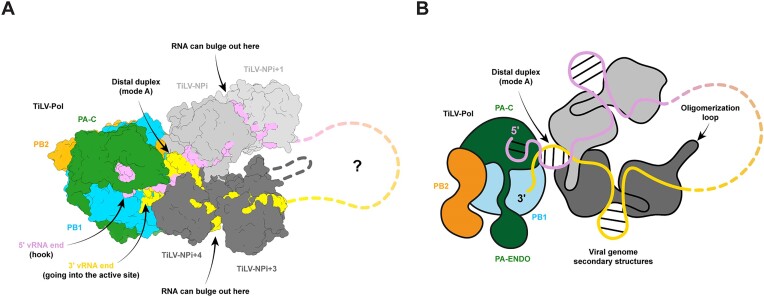
Speculative model of the TiLV polymerase in complex with four proximal TiLV-NPs and viral RNA. (**A**) The A-form RNA observed within the TiLV-NP tetramer (pseudo-C2) was superimposed on the distal duplex observed in TiLV polymerase (TiLV-Pol) pre-initiation state mode A, with the 3′ end going into PB1 active site (PDB: 8PSN). TiLV-NPi + 1 and TiLV-NPi + 3 have been modelled using the TiLV-NP hexamer (pseudo-C6) by superposition on TiLV-NPi and TiLV-NPi + 4, respectively. Modelling of two additional NPs using any TiLV-NP oligomers on TiLV-NPi + 1 and TiLV-NPi + 3 would result in severe clashes suggesting reorganisation perhaps into a double-stranded helical nucleocapsid. The presented model remains hypothetical, and the TiLV nucleocapsid architecture remains unknown. TiLV-Pol subunits are coloured green (PA), blue (PB1), and orange (PB2). TiLV-NPi and TiLV-NPi + 1 are light grey and bind the 5′ end in plum. TiLV-NPi + 3 and TiLV-NPi + 4 are coloured in dark grey and bind the 3′ end in gold. A few clashes, only at flexible loop positions, occur between TiLV-Pol and adjacent TiLV-NPi and TiLV-NPi + 4. (**B**) Schematic of the putative TiLV-Pol/TiLV-NP organisation. Proteins and RNAs are coloured as in panel A. This model is in accordance with the tail-loop association first (Turell *et al.*, 2013) with TiLV-NP oligomerisation loop following the 5′ extremity. At the NP-NP interface, RNA can bulge out and form secondary structures.

While this manuscript was under review, the high-resolution structure of an *in vitro* reconstituted right-handed, anti-parallel, double-helical influenza RNP-like assembly was reported [23]. A complete RNA pathway within FluA/H1N1-NP, as well as the different NP–NP interactions that stabilise the helical assembly are described. This structure was made possible by truncating the probably disordered N-terminal extension of NP, which increased its ability to form rigid nucleocapsid-like assemblies, and through the use of 3′-FAM modified short RNAs. The number of nucleotides binding to NP is found to range from 20 to 24. Comparison of the RNA-bound FluA/H1N1-NP and TiLV-NP structures suggests that the general RNA-binding mode is similar, although the details are different (Supplementary Fig. S9). The RNA directionality is the same, and also consistent with the structure of FluA/H5N1-NP with three nucleotides bound [15]. The RNA path and all previously described RNA-interacting residues largely superimpose with the RNA binding groove observed in TiLV-NP, further supporting the conserved principles of RNA binding within orthomyxovirus RNPs (Supplementary Fig. S9). Furthermore, these observations are consistent with the ‘tail-loop first’ model of NP directionality deduced using oligomerisation defective mutants in cells [33] (Fig. 5; Supplementary Fig. S9).

Nevertheless, several questions remain. Although previous studies have provided low-resolution EM images of purified TiLV RNPs [24], their quality precluded further characterisation, and the helical parameters remain unknown (Fig. 5). Assuming TiLV-RNPs adopts an anti-parallel superhelix like influenza, it is tempting to suggest that the circularised TiLV-NP pentamers and hexamers may correspond to the loop found at one extremity of the RNP or could, perhaps, correspond to a nascent RNP formed within the replication complex once the RNA product begins to bulge out [10,11]. In the pseudo-C2 structure, the duplex A-form RNA that splays out into single-stranded 3′ and 5′ extensions that bind to adjacent NPs, could also mimic the polymerase proximal promoter duplex separating into incoming and outgoing template strands, each binding an NP at the start of the RNP superhelix. Indeed, superposition of the TiLV-NP A-form RNA onto the distal duplex seen in TiLV polymerase [4] shows that both proximal NPs avoid severe steric clashes with TiLV-Pol (Fig. 5). However, *in vitro* reconstitution of TiLV RNPs, as recently achieved for FluA [21], or cryo-ET on purified viral RNPs, will be necessary to provide further insights into TiLV RNP morphology and dynamics during RNA synthesis.

Finally, despite having mapped the previously described mutations introduced using a yeast-three hybrid system onto our TiLV-NP structures and showing consistency of results, *in cellulo* functional studies remain challenging due to the lack of an established TiLV minigenome system. Very recently, a reverse genetics system for TiLV rescue has been described [40], the first step to more flexible laboratory tools to probe the mechanism of TiLV replication and establishing a platform for investigating antiviral strategies targeting either the NP–NP, NP–RNA interfaces, or the polymerase function.

To conclude, studying phylogenetically divergent viruses such as TiLV offers valuable insights into conserved or divergent mechanisms of transcription, replication, and genome packaging within the *Articulavirales* order.

## Supplementary Material

gkaf112_Supplemental_Files

## Data Availability

The data that support this study are available from the corresponding authors upon reasonable request. The EM maps and co-ordinates generated in this study have been deposited in the Electron Microscopy Data Bank and the Protein Data Bank (summarised in Supplementary Tables 1-4): TiLV-NP pentamer (pseudo-C5) (local refinement around 2 TiLV-NPs) PDB ID 9HBR EMD-52027. TiLV-NP pentamer (pseudo-C5) PDB ID 9HBT EMD-52029. TiLV-NP tetramer (pseudo-C2) (local refinement around 2 TiLV-NPs) PDB ID 9HBU EMD-52030. TiLV-NP tetramer (pseudo-C2) PDB ID 9HBS EMD-52028. TiLV-NP tetramer (pseudo-C4) (local refinement around 2 TiLV-NPs) PDB ID 9HBV EMD-52031. TiLV-NP tetramer (pseudo-C4) PDB ID 9HBW EMD-52032. TiLV-NP hexamer (pseudo-C6) (local refinement around 2 TiLV-NPs) PDB ID 9HBX EMD-52033. TiLV-NP hexamer (pseudo-C6) (local refinement around 3 TiLV-NPs) PDB ID 9HBY EMD-52035. TiLV-NP hexamer (pseudo-C6) PDB ID 9HBZ EMD-52035. Source data are provided in a supplementary file associated with this paper.

## Notes

Present address: Institute of Science and Technology Austria (ISTA), Klosterneuburg, Austria
