# Supplementary material for: Structure of the tilapia lake virus nucleoprotein bound to RNA
Source: Nucleic Acids Res. 2025 Feb 24;53(4):gkaf112. doi: 10.1093/nar/gkaf112 (PMC11850232; doi:10.1093/nar/gkaf112)
Supplement: gkaf112_Supplemental_Files [file gkaf112_supplemental_files.zip › SUPPLEMENTARY-FIGURES-240125.pdf]

SUPPLEMENTARY FIGURE 1

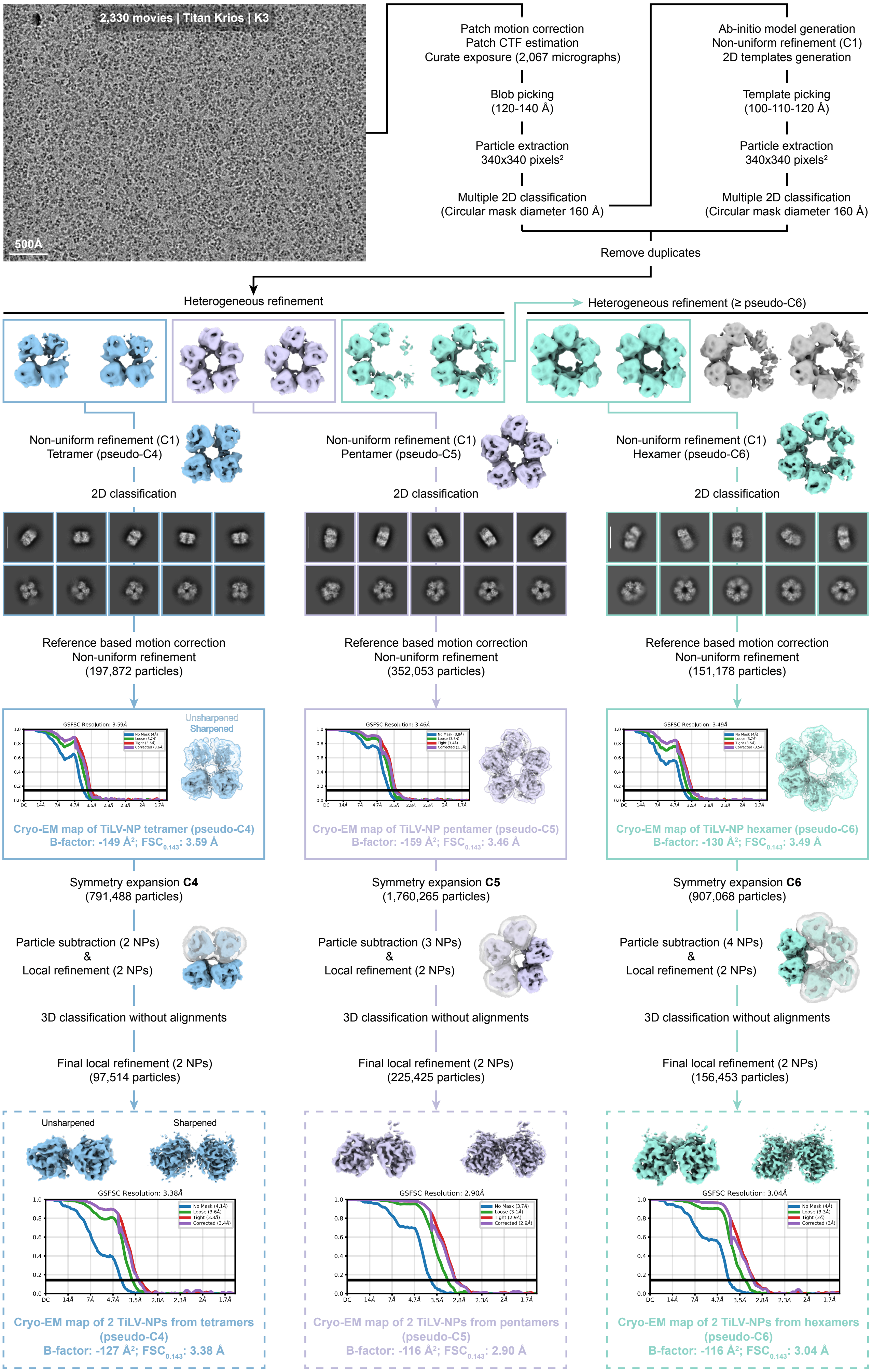

Supplementary Figure 1. General cryo-EM image processing strategy applied to obtain TiLV-NP structures (tetrameric pseudo-C4, pentameric pseudo-C5, and hexameric pseudo-C6).
Schematics of the image processing strategy used with the data collected on a Titan Krios equipped with a Gatan K3 direct electron detector mounted on a Gatan Bioquantum energy filter. Representative realigned micrograph (-3 μM defocus, low-pass filtered at 5 Å, scale bar = 500 Å), 2D class averages (scale bar = 120 Å), and 3D maps are displayed. Fourier shell correlation curves (FSC) are shown, and the overall resolution based on the FSC0.143 criteria indicated.

SUPPLEMENTARY FIGURE 2

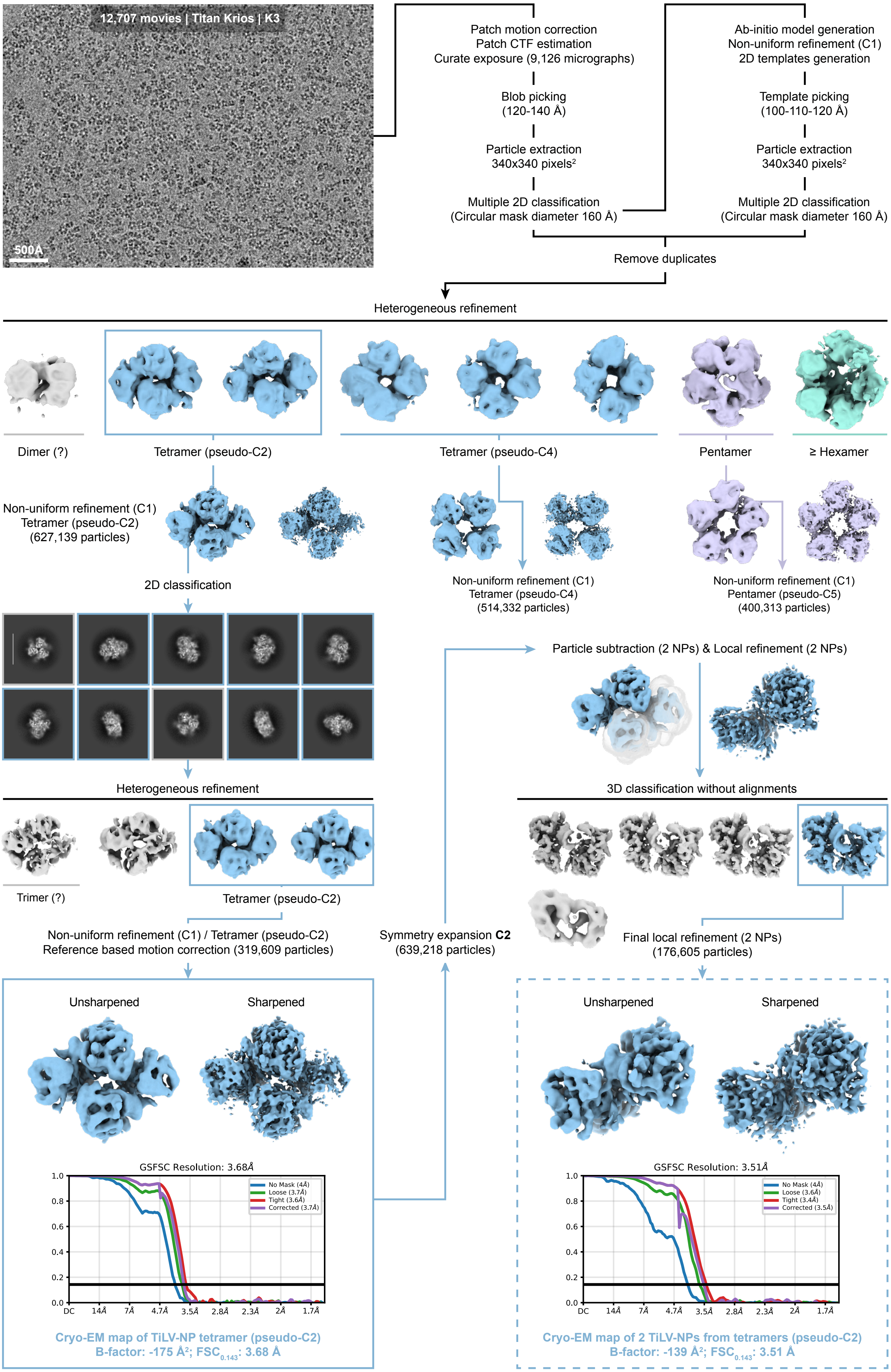

Supplementary Figure 2. General cryo-EM image processing strategy applied to obtain TiLV-NP structures (tetrameric pseudo-C2). Schematics of the image processing strategy used with the data collected on a Titan Krios equipped with a Gatan K3 direct electron detector mounted on a Gatan Bioquantum energy filter. Representative realigned micrograph (-3 µM defocus, low-pass filtered at 5 Å, scale bar = 500 Å), 2D class averages (scale bar = 120 Å), and 3D maps are displayed. Fourier shell correlation curves (FSC) are shown, and the overall resolution based on the FSC0.143 criteria indicated.

SUPPLEMENTARY FIGURE 3

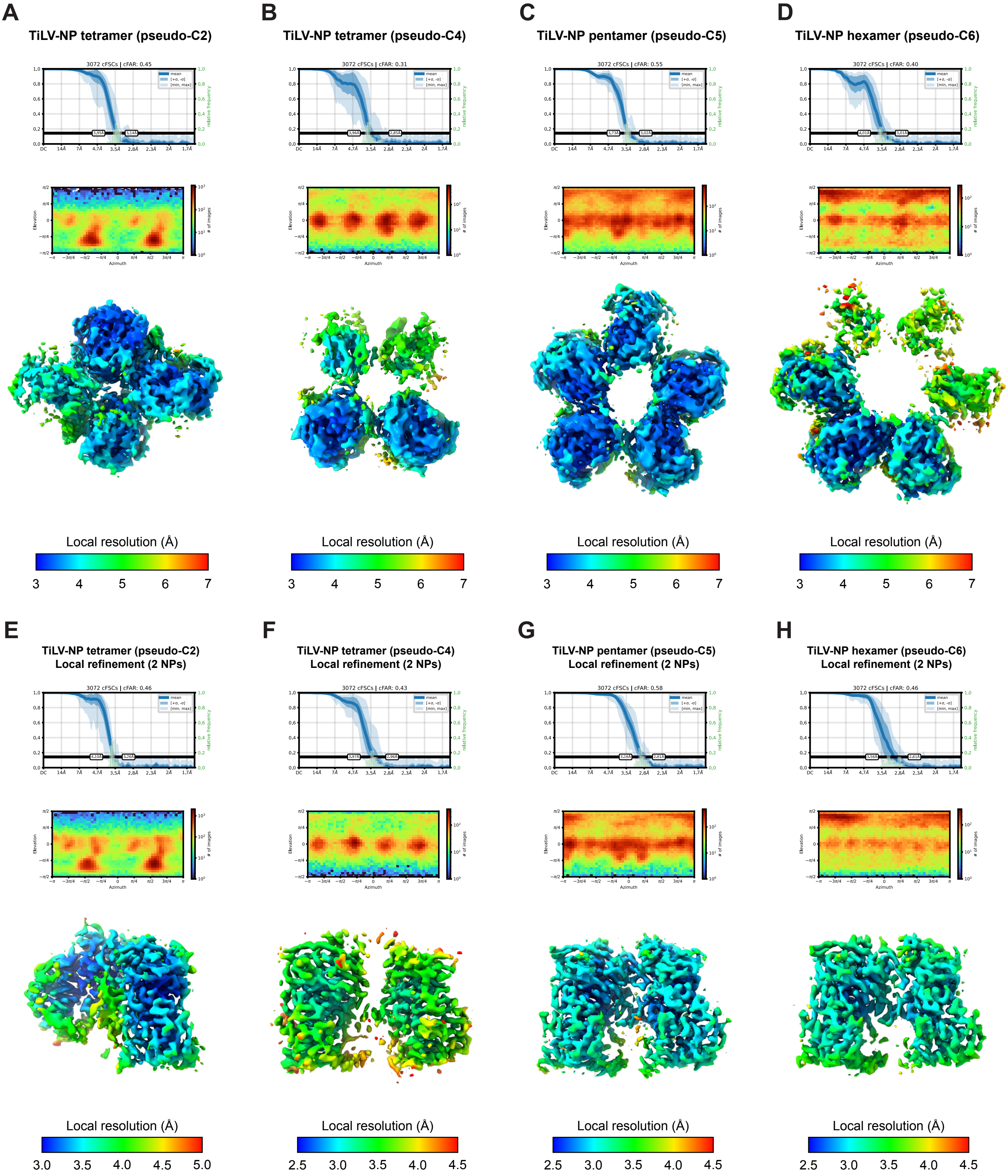

Supplementary Figure 3. 3D-FSCs, orientation distribution and local resolution of each TiLV-NP structures.
A-D. 3D-FSCs, orientation distribution and local resolution of each complete TiLV-NP structures (A) pseudo-C2; (B) pseudo-C4; (C) pseudo-C5; (D) pseudo-C6.
E-H. 3D-FSCs, orientation distribution and local resolution of each local refinement around two TiLV-NPs for each oligomer (E) pseudo-C2; (F) pseudo-C4; (G) pseudo-C5; (H) pseudo-C6.

SUPPLEMENTARY FIGURE 4

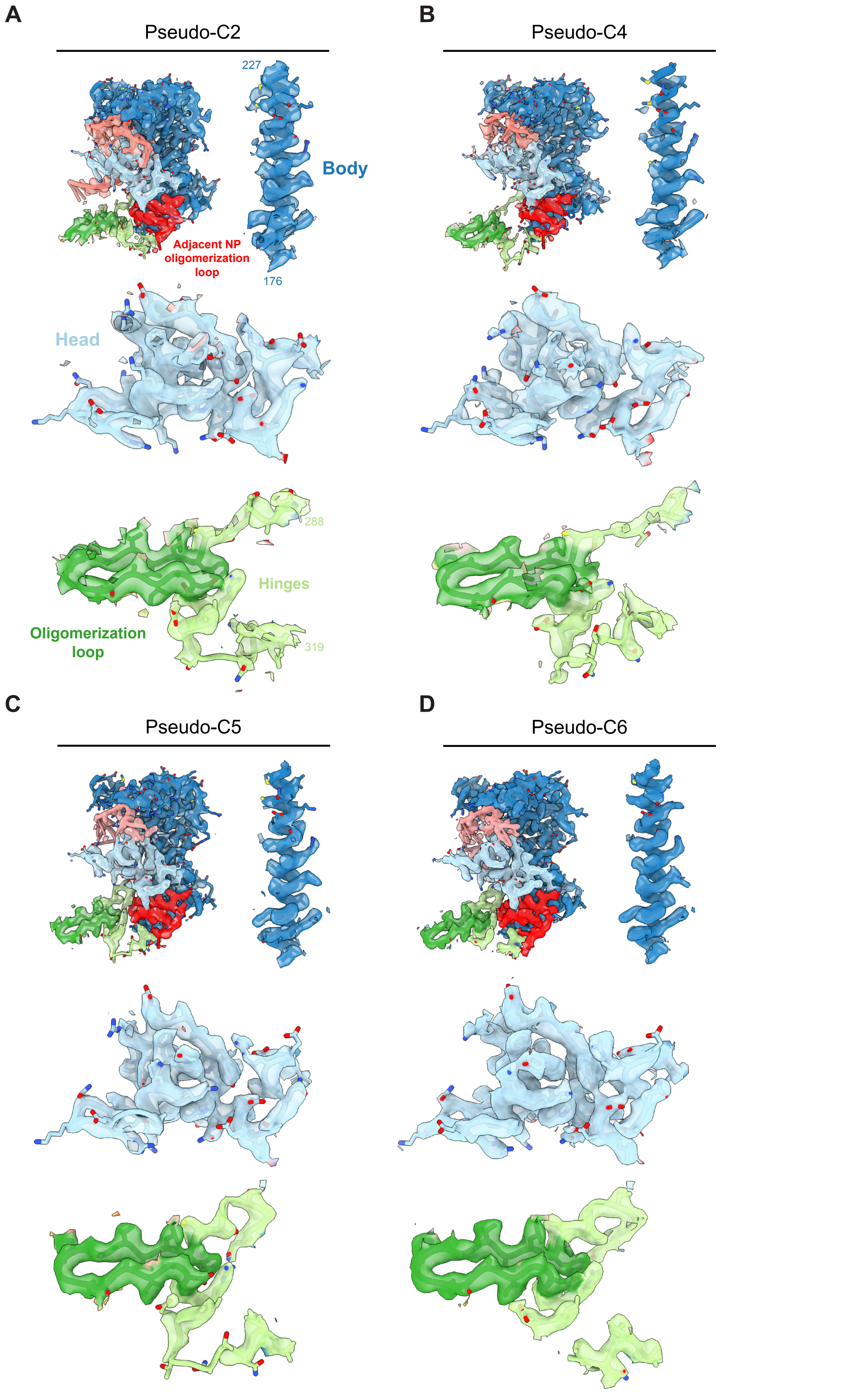

Supplementary Figure 4. Cryo-EM map quality of TiLV-NP structures.
A-D. Extracted coulomb potential of a single TiLV-NP from the local refinement around two NPs from the (A) pseudo-C2 tetramer, (B) pseudo-C4 tetramer, (C) pseudo-C5 pentamer, (D) pseudo-C6 hexamer maps. Additional EM densities are shown for the body, head, hinges, and the oligomerization loop, with side chains clearly visible.

SUPPLEMENTARY FIGURE 5

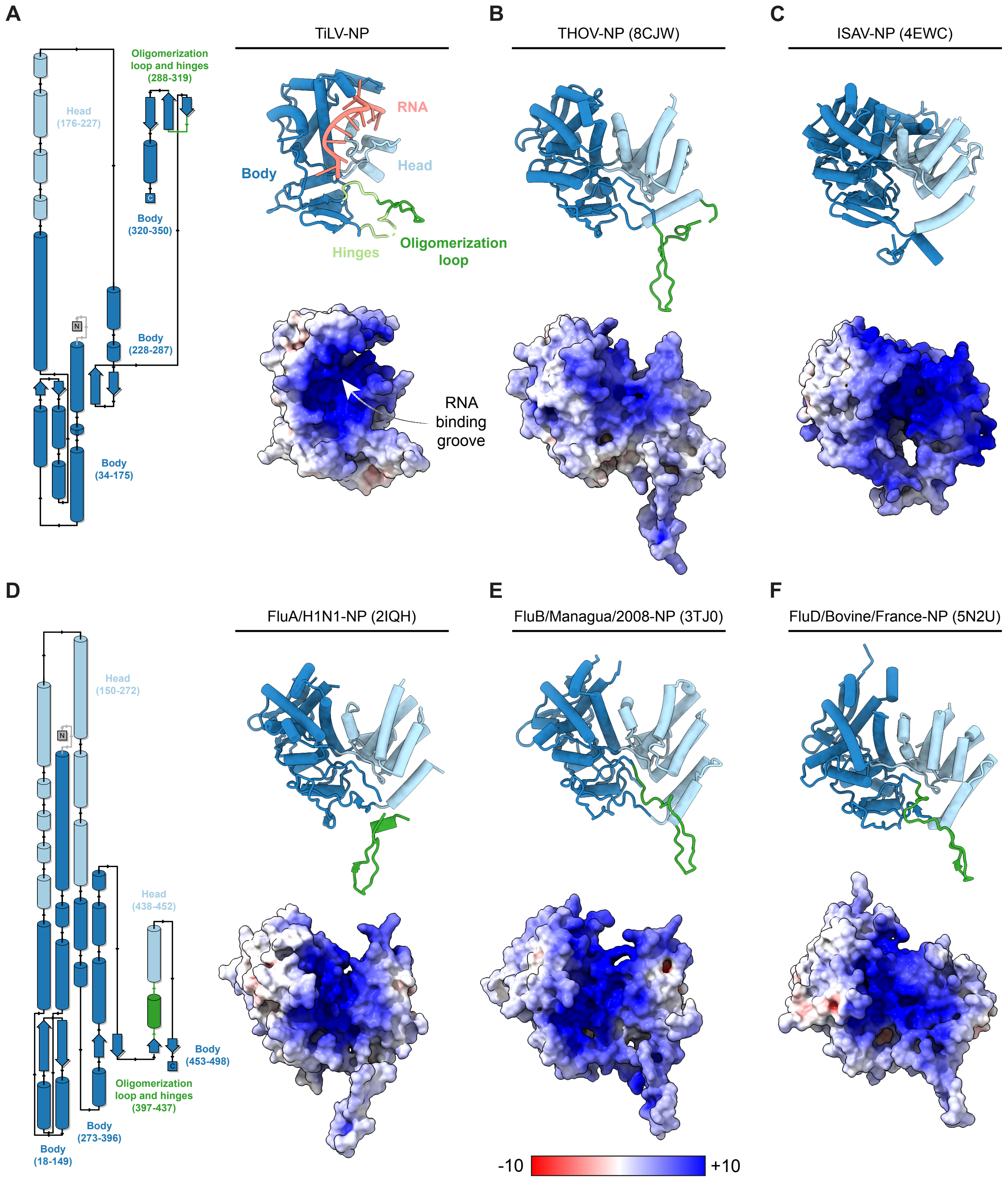

Supplementary Figure 5. Structural comparison of TiLV-NP monomer with other orthomyxo(-like) viruses NPs.
A. Left: TiLV-NP topology diagram calculated using PDBsum. Alpha-helices and beta-strands are shown as cylinders and arrows, respectively. Secondary structures are coloured as in Fig. 2. N-terminus (N) and C-terminus (C) are indicated. Top right: Cartoon representation of RNA-bound TiLV-NP. TiLV-NP domains and RNA are coloured as in Fig. 2. Bottom right: Surface representation of RNA-bound TiLV-NP coloured based on electrostatic potential.
B. Top: Cartoon representation of THOV-NP (PDB 8CJW). THOV-NP domains are coloured as TiLV-NP. Bottom: Surface representation of THOV-NP coloured based on electrostatic potential.
C. Top: Cartoon representation of ISAV-NP (PDB 4EWC). ISAV-NP domains are coloured as TiLV-NP. Bottom: Surface representation of ISAV-NP coloured based on electrostatic potential.
D. Left: FluA/H1N1-NP topology diagram calculated using PDBsum. Alpha-helices and beta-strands are shown as cylinders and arrows, respectively. Secondary structures are coloured as in Fig. 2. N-terminus (N) and C-terminus (C) are indicated. Top right: Cartoon representation of FluA/H1N1-NP (PDB 2IQH). FluA/H1N1-NP domains are coloured as TiLV-NP. Bottom right: Surface representation of FluA/H1N1-NP coloured based on electrostatic potential.
E. Top: Cartoon representation of FluB/Managua/2008-NP (PDB 3TJ0). FluB/Managua/2008-NP domains are coloured as TiLV-NP. Bottom: Surface representation of FluB/Managua/2008-NP coloured based on electrostatic potential.
F. Top: Cartoon representation of FluD/Bovine/France-NP (PDB 5N2U). FluD/Bovine/France-NP domains are coloured as TiLV-NP. Bottom: Surface representation of FluD/Bovine/France-NP coloured based on electrostatic potential.

SUPPLEMENTARY FIGURE 6

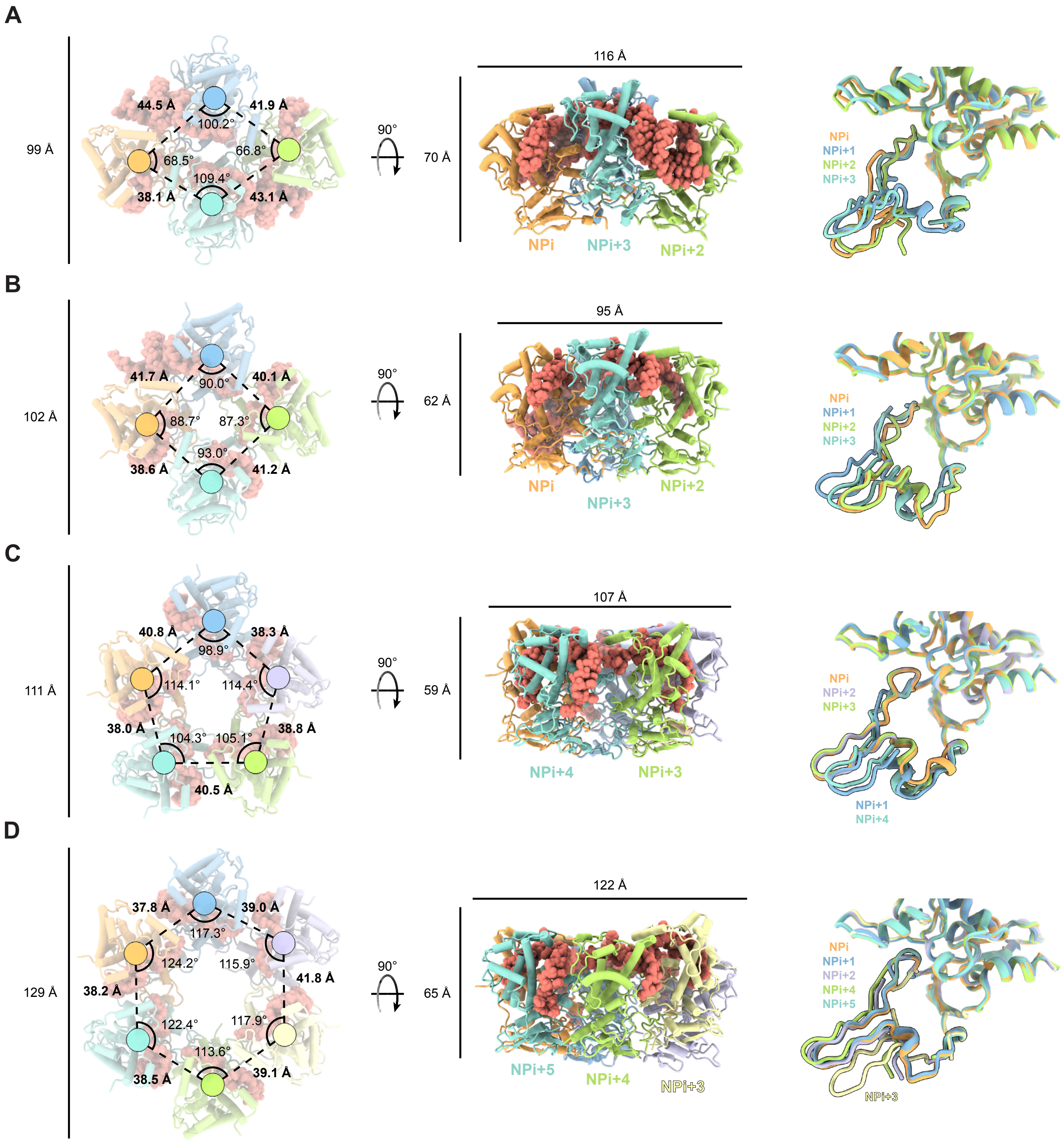

Supplementary Figure 6. Pseudo-symmetrical arrangement of TiLV-NP oligomers.
A-D. Cartoon representation of (A) TiLV-NP tetramer (pseudo-C2), (B) TiLV-NP tetramer (pseudo-C4), (C) TiLV-NP pentamer (pseudo-C5), (D) TiLV-NP hexamer (pseudo-C6), with NP centroids shown as spheres. Oligomer dimensions, inter-NP distances and angles are indicated. Superposition of individual NPs for each oligomer highlights flexibility in the hinges and the oligomerization loop, underlined with a black line.

SUPPLEMENTARY FIGURE 7

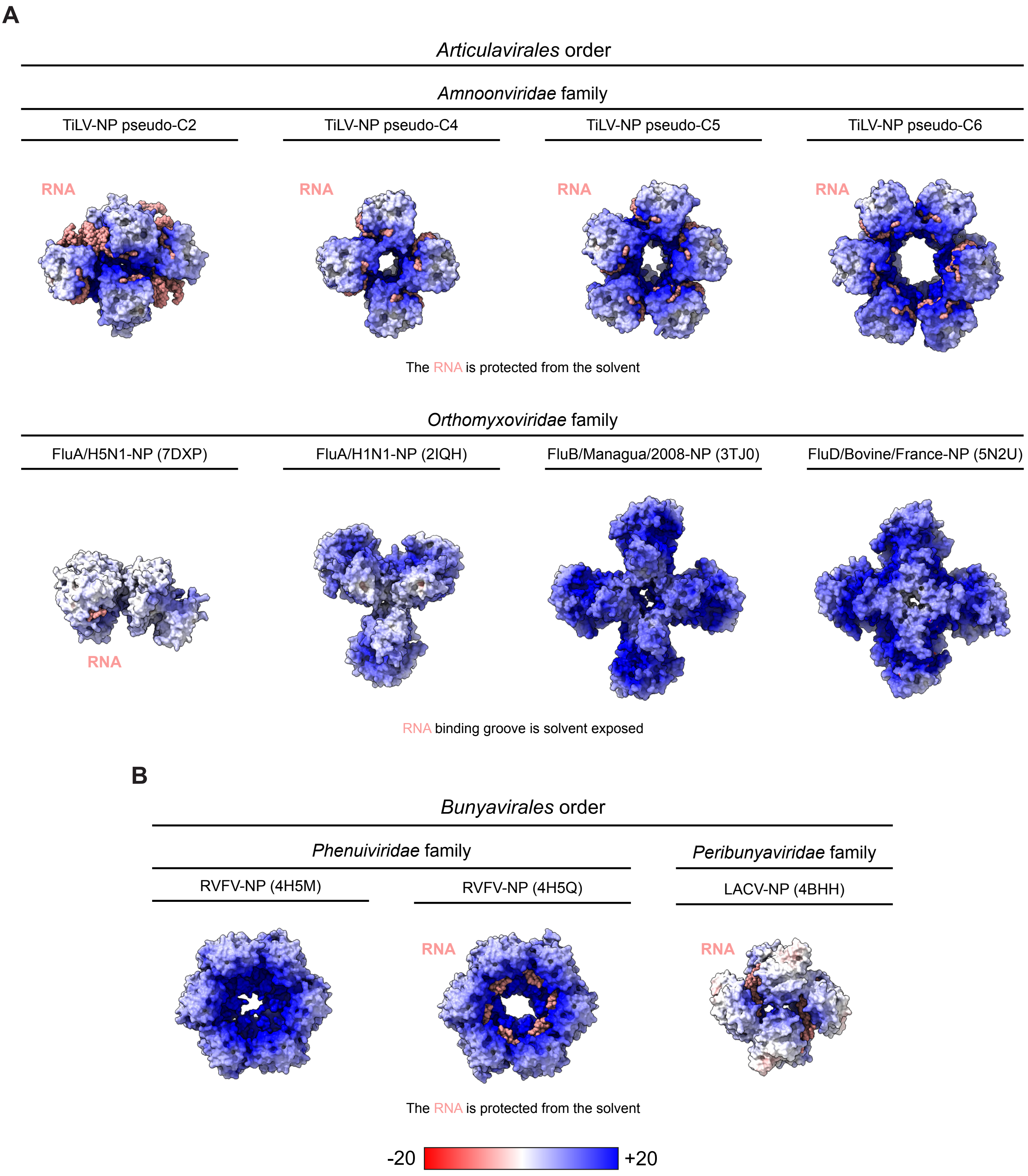

Supplementary Figure 7. Structural comparison of TiLV-NP oligomers with other orthomyxo(-like)- and bunya- viruses NPs.
A. Surface representations of NPs from orthomyxo(-like) viruses, coloured based on electrostatic potential. The top row shows RNA-bound TiLV-NP structures determined by cryo-EM in this study. The bottom row shows X-ray structures of FluA/H5N1-NP (PDB 7DXP), FluA/H1N1-NP (PDB 2IQH), FluB/Managua/2008-NP (PDB 3TJ0), FluD/Bovine/France-NP (PDB 5N2U). In TiLV-NP oligomers, the RNA is protected from the solvent, whereas in the Flu-NPs X-ray structures, the RNA binding groove is solvent exposed. The RNA is displayed as spheres, coloured in salmon.
B. Surface representations of bunyavirus NPs X-ray structures (Rift Valley Fever virus "RVFV", PDB 4H5M / 4H5Q; La Crosse virus "LACV", PDB 4BHH), coloured based on electrostatic potential. The RNA binding groove is protected from the solvent.

SUPPLEMENTARY FIGURE 8

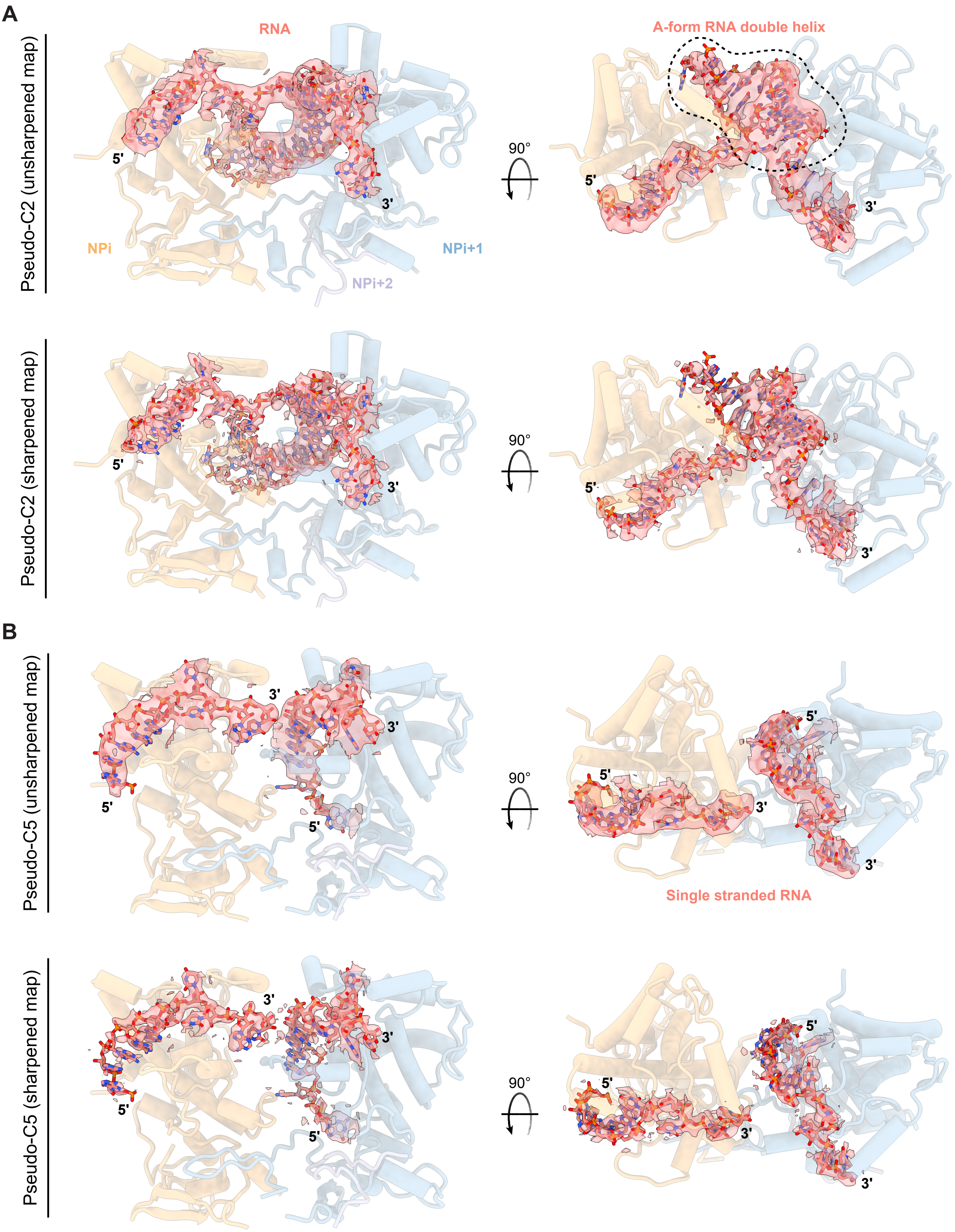

Supplementary Figure 8. Cryo-EM map quality of the RNA within TiLV-NP structures.
A. RNA coulomb potential extracted from the local refinement around two TiLV NPs from the pseudo-C2 tetramer map. The RNA is coloured in salmon, NPi orange, NPi+1 blue and NPi+2 mauve. The 5′ and 3′ ends are annotated. Both unsharpened and sharpened maps are shown.
B. RNA coulomb potential extracted from the local refinement around two TiLV NPs from the pseudo-C5 pentamer map. The RNA is coloured in salmon, NPi orange, NPi+1 blue and NPi+2 mauve. The 5′ and 3′ ends are annotated. Both unsharpened and sharpened maps are shown.

SUPPLEMENTARY FIGURE 9

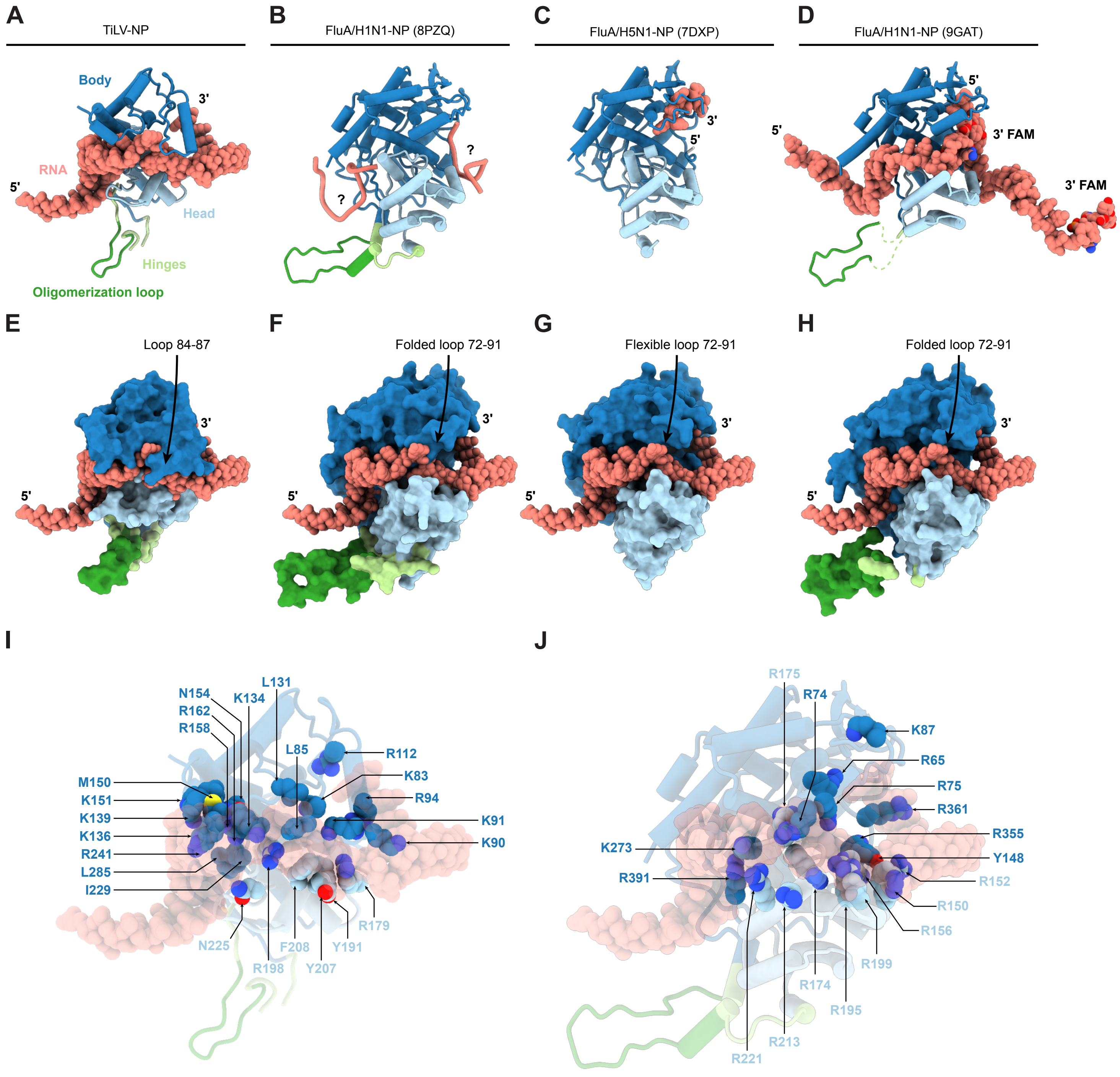

Supplementary Figure 9. Superposition of TiLV-NP RNA on influenza NPs.
A. Cartoon representation of RNA-bound TiLV-NP. All RNA conformations observed in the TiLV-NP pseudo-C2 and pseudo-C5 structures are superimposed (see Fig. 4B, F). The RNAs are shown as spheres. TiLV-NP domains are coloured as in Fig 2. The 5′ and 3′ ends are annotated.
B. Cartoon representation of FluA/H1N1-NP (PDB 8PZQ). Only one NP is displayed (chain A). The RNA phosphate backbone is shown as liquorice as the RNA polarity and base position are unknown. Flu-NP domains are coloured as in Fig 2.
C. Cartoon representation of FluA/H5N1-NP (PDB 7DXP). Only one NP is displayed (chain B). The RNA with methylated 2′ OHs is shown as spheres. The 5′ and 3′ ends are annotated. Flu-NP domains are coloured as in Fig 2.
D. Cartoon representation of FluA/H1N1-NP (PDB 9GAT). Only one NP is displayed (chain J). The two RNAs binding to one NP are shown as spheres, with 3′-FAMs coloured by heteroatoms. The 5′ and 3′ ends are annotated. Flu-NP domains are coloured as in Fig 2.
E. Similar to A, but TiLV-NP is displayed as surface.
F. Superposition of TiLV-NP RNAs onto FluA/H1N1-NP (PDB 8PZQ). TiLV-NP RNAs follow the Flu-NP RNA binding groove. One "clash" is next to the Flu-NP folded loop 72-91, which is flexible in FluA/H5N1-NP (PDB 7DXP).
G. Superposition of TiLV-NP RNAs onto FluA/H1N1-NP (PDB 9GAT). TiLV-NP RNAs follow the Flu-NP RNA binding groove. The RNA polarity is conserved.
H. Superposition of TiLV-NP RNAs onto FluA/H5N1-NP (PDB 7DXP). TiLV-NP RNAs follow the Flu-NP RNA binding groove. The RNA polarity is conserved with the 3 methylated nucleotides observed in FluA/H5N1-NP (PDB 7DXP) X-ray structure.
I. Summary of TiLV-NP residues involved in RNA interactions. TiLV-NP and all superimposed RNA conformations observed in the TiLV-NP pseudo-C2 and pseudo-C5 structures are transparent. Key interacting residues are displayed as spheres and coloured according to panel A.
J. Summary of residues in Flu-NP reported to interact with RNA. Flu-NP and all superimposed RNA conformations observed in the TiLV-NP pseudo-C2 and pseudo-C5 structures are transparent. Key interacting residues are displayed as spheres and coloured according to panel A.

SUPPLEMENTARY FIGURE 10

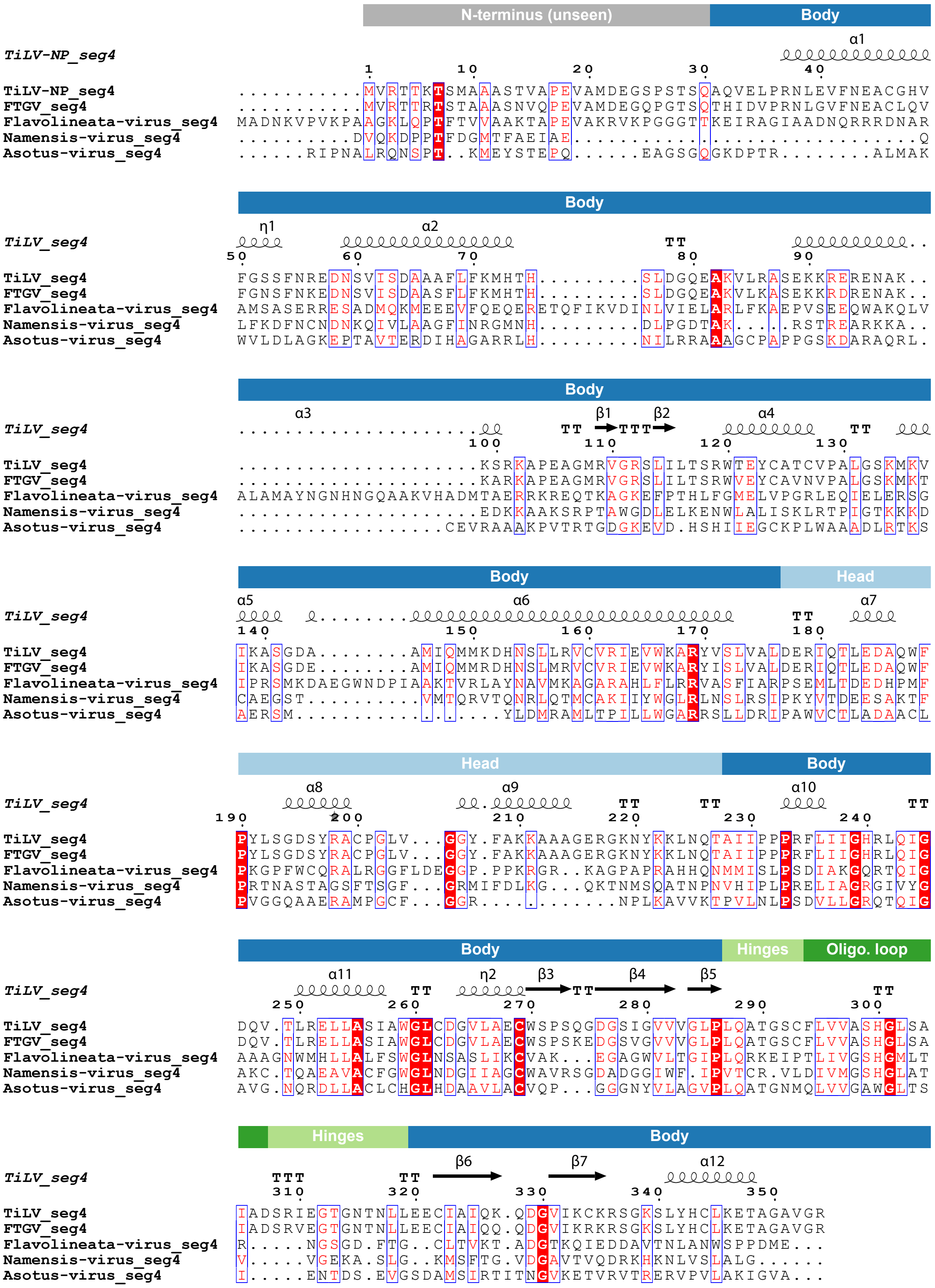

Supplementary Figure 10. Multiple sequence alignment of TiLV-NP segment 4 with corresponding segment 4 from Fancy tailed guppy virus (Amnoonviridae family), Flavolineata, Namensis, and Asotus viruses (not classified).

TiLV-NP secondary structures are shown and numbered. Domain positions are indicated. Multiple sequence alignment was performed using MUSCLE {Edgar, 2004 #2609} with TiLV-NP (segment 4; GenBank: AOE22901.1), Fancy tailed guppy virus (FTGV; segment 4; GenBank: WWU01869.1). Flavolineata, Namensis and Asotus virus putative NP sequences were derived from {Turnbull, 2020 #2134}.

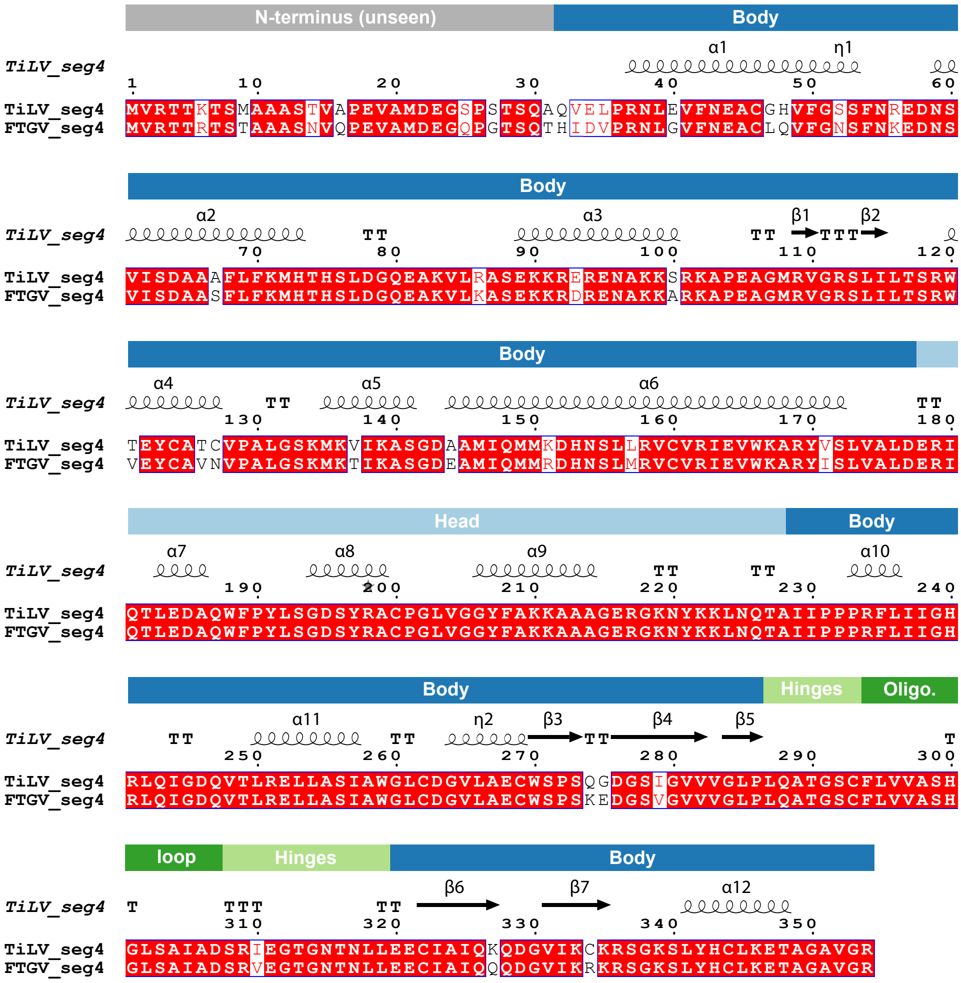
N-terminus (unseen)
Body
TiLV_seg4
α1 η1
TiLV_seg4 MVRTTKTSMAAASTVAPEVAMDEGSPSTSQAQVELPRNLEVFNEACGHVFGSSFNREDNS
FTGV_seg4 MVRTTRTSTAAASNVQPEVAMDEGQPGTSQTHIDVPRNLGVFNEACLQVFGNSFNKEDNS
Body
α2 α3 β1 β2
TiLV_seg4 VISDAAAFLFKMHTHSLDGQEAKVLRASEKKRERENAKKSRKAPEAGMRVGRSLILTSRW
FTGV_seg4 VISDAASFLFKMHTHSLDGQEAKVLKASEKKRDRENAKKARKAPEAGMRVGRSLILTSRW
Body
α4 α5 α6
TiLV_seg4 TEYCATCVPALGSKMKVIKASGDAAMIQMMKDHNSLLRVCVRIEVWKARYVSLVALDERI
FTGV_seg4 VEYCAVNVPALGSKMKTIKASGDEAMIQMMRDHNSLMRVCVRIEVWKARYISLVALDERI
Head
Body
α7 α8 α9 α10
TiLV_seg4 QTLEDAQWFPYLSGDSYRACPGLVGGYFAKKAAAGERGKNYKKLNQTAIIPPPRFLIIGH
FTGV_seg4 QTLEDAQWFPYLSGDSYRACPGLVGGYFAKKAAAGERGKNYKKLNQTAIIPPPRFLIIGH
Body
Hinges
Oligo.
α11 η2 β3 β4 β5
TiLV_seg4 RLQIGDQVTLRELLASIAWGLCDGVLAECWSPSQGDGSIGVVVGLPLQATGSCFLVVASH
FTGV_seg4 RLQIGDQVTLRELLASIAWGLCDGVLAECWSPSKEDGSVGVVVGLPLQATGSCFLVVASH
loop
Hinges
Body
β6 β7 α12
TiLV_seg4 GLSAIADSRIEGTGNTNLLEECIAIQKQDGVIKCKRSGKSLYHCLKETAGAVGR
FTGV_seg4 GLSAIADSRVEGTGNTNLLEECIAIQQQDGVIKRKRSGKSLYHCLKETAGAVGR
