# Supplementary material for: Structure of the tilapia lake virus nucleoprotein bound to RNA
Source: Nucleic Acids Res. 2025 Feb 24;53(4):gkaf112. doi: 10.1093/nar/gkaf112 (PMC11850232; doi:10.1093/nar/gkaf112)
Supplement: gkaf112_Supplemental_Files [file gkaf112_supplemental_files.zip › Supplementary-Tables-1-4-TiLV-NP.pdf]

Supplementary Table 1. Cryo-EM data collection, refinement and validation statistics for the RNA-bound TiLV-NP pseudo-C5 oligomer structures.

Sample	TiLV-NP with 40-mer vRNA<br>2 NPs from pentamer	TiLV-NP with 40-mer vRNA<br>Complete pentamer
PDB ID	9HBR	9HBT
EMDB ID	EMD-52027	EMD-52029
**Data collection and processing**		
Microscope	ThermoFisher TEM Titan Krios G3	
Voltage (kV)	300	
Camera	Gatan K3 / BioQuantum EF	
Magnification	105,000	
Nominal defocus range (µm)	-0.8 / -2.0	
Electron exposure (e–/Å^2)	40	
Pixel size (Å)	0.822	
Initial micrographs (no.)	2330	
Final micrographs (no.)	2067	
**Refinement**		
Particles per class (no.)	225,425	352,053
Map resolution (Å), 0.143 FSC	2.90	3.46
Model resolution (Å), 0.5 FSC	3.0	3.7
Map sharpening *B* factor (Å^2)	-116	-127
Map versus model cross-correlation (CCmask)	0.8105	0.6841<br>(2 subunits poor density)
**Model composition**		
Non-hydrogen atoms	5268	13417
Protein residues	625	1581
RNA nts	22	57
**Mean *B* factors (Å^2)**		
Protein	69.63	58.98
RNA	125.79	97.53
**RMS deviations**		
Bond lengths (Å)	0.003	0.002
Bond angles (°)	0.397	0.382
**Validation**		
MolProbity score	1.23	1.50
All-atom clash score	1.54	3.67
Poor rotamers (%)	2.76	2.81
**Ramachandran plot**		
Favored (%)	98.21	98.28
Allowed (%)	1.79	1.72
Outliers (%)	0.0	0.0

Supplementary Table 2. Cryo-EM data collection, refinement and validation statistics for the RNA-bound TiLV-NP pseudo-C6 oligomer structures.

**Sample**	**TiLV-NP with 40-mer vRNA (2 NPs from hexamer)**	**TiLV-NP with 40-mer vRNA (3 NPs from hexamer)**	**TiLV-NP with 40-mer vRNA (Complete hexamer)**
PDB ID	9HBX	9HBY	9HBZ
EMDB ID	EMD-52033	EMD-52034	EMD-52035
**Data collection and processing**			
Microscope	ThermoFisher TEM Titan Krios G3		
Voltage (kV)	300		
Camera	Gatan K3 / BioQuantum EF		
Magnification	105,000		
Nominal defocus range (µm)	-0.8 / -2.0		
Electron exposure (e–/Å^2)	40		
Pixel size (Å)	0.822		
Initial micrographs (no.)	2330		
Final micrographs (no.)	2067		
**Refinement**			
Particles per class (no.)	156,453		151,178
Map resolution (Å), 0.143 FSC	3.06	3.1	3.49
Model resolution (Å), 0.5 FSC	3.2	3.3	4.0
Map sharpening *B* factor (Å^2)	-116	-131	-130
Map versus model cross-correlation (CCmask)	0.7912	0.7815	0.6051 (2 subunits poor density)
**Model composition**			
Non-hydrogen atoms	5291	8025	15792
Protein residues	630	951	1881
RNA nts	22	35	54
**Mean *B* factors (Å^2)**			
Protein	68.83	78.40	61.29
RNA	130.20	132.52	93.87
**RMS deviations**			
Bond lengths (Å)	0.003	0.002	0.002
Bond angles (°)	0.431	0.390	0.444
**Validation**			
MolProbity score	1.51	1.35	1.45
All-atom clash score	3.36	3.48	3.95
Poor rotamers (%)	1.77	1.04	2.23
**Ramachandran plot**			
Favored (%)	96.78	96.81	98.17
Allowed (%)	3.22	3.19	1.83
Outliers (%)	0.0	0.0	0.0

Supplementary Table 3. Cryo-EM data collection, refinement and validation statistics for the RNA-bound TiLV-NP pseudo-C4 oligomer structures.

Sample	TiLV-NP with 40-mer vRNA (2 NPs from pseudo-C4 tetramer)	TiLV-NP with 40-mer vRNA (Complete pseudo-C4 tetramer)
PDB ID	9HBV	9HBW
EMDB ID	EMD-52031	EMD-52032
**Data collection and processing**		
Microscope	ThermoFisher TEM Titan Krios G3	
Voltage (kV)	300	
Camera	Gatan K3 / BioQuantum EF	
Magnification	105,000	
Nominal defocus range (µm)	-0.8 / -2.0	
Electron exposure (e–/Å^2)	40	
Pixel size (Å)	0.822	
Initial micrographs (no.)	2330	
Final micrographs (no.)	2067	
**Refinement**		
Particles per class (no.)		
Map resolution (Å), 0.143 FSC	3.38	3.59
Model resolution (Å), 0.5 FSC	3.6	4.2
Map sharpening *B* factor (Å^2)	-127	-149
Map versus model cross-correlation (CCmask)	0.7483	0.5831 (2 subunits poor density)
**Model composition**		
Non-hydrogen atoms	5450	10782
Protein residues	629	1259
RNA nts	30	54
**Mean *B* factors (Å^2)**		
Protein	76.16	74.49
RNA	157.81	129.11
**RMS deviations**		
Bond lengths (Å)	0.002	0.003
Bond angles (°)	0.389	0.448
**Validation**		
MolProbity score	1.52	1.38
All-atom clash score	3.75	3.74
Poor rotamers (%)	2.95	1.57
**Ramachandran plot**		
Favored (%)	98.07	97.67
Allowed (%)	1.93	2.33
Outliers (%)	0.0	0.0

Supplementary Table 4. Cryo-EM data collection, refinement and validation statistics for the RNA-bound TiLV-NP pseudo-C2 oligomer structures.

Sample	TiLV-NP with 40-mer vRNA (2 NPs from pseudo-C2 tetramer)	TiLV-NP with 40-mer vRNA (Complete pseudo-C2 tetramer)
PDB ID	9HBU	9HBS
EMDB ID	EMD-52030	EMD-52028
**Data collection and processing**		
Microscope	ThermoFisher TEM Titan Krios G3	
Voltage (kV)	300	
Camera	Gatan K3 / BioQuantum EF	
Magnification	105,000	
Nominal defocus range (µm)	-0.8 / -2.0	
Electron exposure (e–/Å^2)	50	
Pixel size (Å)	0.822	
Initial micrographs (no.)	12,707	
Final micrographs (no.)	9126	
**Refinement**		
Particles per class (no.)	176,605	319,609
Map resolution (Å), 0.143 FSC	3.51	3.68
Model resolution (Å), 0.5 FSC	3.7	4.0
Map sharpening *B* factor (Å^2)	-139	-175
Map versus model cross-correlation (CCmask)	0.7530	0.6426
**Model composition**		
Non-hydrogen atoms	5593	11165
Protein residues	629	1252
RNA nts	37	74
**Mean *B* factors (Å^2)**		
Protein	46.12	57.15
RNA	94.51	111.59
**RMS deviations**		
Bond lengths (Å)	0.002	0.003
Bond angles (°)	0.415	0.449
**Validation**		
MolProbity score	1.56	1.58
All-atom clash score	3.31	4.15
Poor rotamers (%)	1.97	1.57
**Ramachandran plot**		
Favored (%)	96.62	96.45
Allowed (%)	3.22	3.47
Outliers (%)	0.16	0.08
